# Comparative transcriptome and DNA methylation analysis in temperature-sensitive genic male sterile wheat BS366

**DOI:** 10.1186/s12864-021-08163-3

**Published:** 2021-12-20

**Authors:** Yong-jie Liu, Dan Li, Jie Gong, Yong-bo Wang, Zhao-bo Chen, Bin-shuang Pang, Xian-chao Chen, Jian-gang Gao, Wei-bing Yang, Feng-ting Zhang, Yi-miao Tang, Chang-ping Zhao, Shi-qing Gao

**Affiliations:** 1 Beijing Engineering Research Center for Hybrid Wheat, Beijing Academy of Agriculture and Forestry Sciences, Beijing, 100097 China; 2 The Municipal Key Laboratory of the Molecular Genetics of Hybrid Wheat, Beijing, 100097 China

**Keywords:** *Triticum aestivum* L., Pollen, Temperature-sensitive genic male sterility (TGMS), Transcriptome, DNA methylation

## Abstract

**Background:**

Known as the prerequisite component for the heterosis breeding system, the male sterile line determines the hybrid yield and seed purity. Therefore, a deep understanding of the mechanism and gene network that leads to male sterility is crucial. BS366, a temperature-sensitive genic male sterile (TGMS) line, is male sterile under cold conditions (12 °C with 12 h of daylight) but fertile under normal temperature (20 °C with 12 h of daylight).

**Results:**

During meiosis, BS366 was defective in forming tetrads and dyads due to the abnormal cell plate. During pollen development, unusual vacuolated pollen that could not accumulate starch grains at the binucleate stage was also observed. Transcriptome analysis revealed that genes involved in the meiotic process, such as sister chromatid segregation and microtubule-based movement, were repressed, while genes involved in DNA and histone methylation were induced in BS366 under cold conditions. MethylRAD was used for reduced DNA methylation sequencing of BS366 spikes under both cold and control conditions. The differentially methylated sites (DMSs) located in the gene region were mainly involved in carbohydrate and fatty acid metabolism, lipid metabolism, and transport. Differentially expressed and methylated genes were mainly involved in cell division.

**Conclusions:**

These results indicated that the methylation of genes involved in carbon metabolism or fatty acid metabolism might contribute to male sterility in BS366 spikes, providing novel insight into the molecular mechanism of wheat male sterility.

**Supplementary Information:**

The online version contains supplementary material available at 10.1186/s12864-021-08163-3.

## Background

Known as an environmentally sustainable and safe way to feed the increasing global population, heterosis has been shown to increase the crop yield by 3.5–15% [1]. It has also been successfully implemented in crops such as maize [2] and rice [3]; however, hybrid wheat is only grown on less than 0.2% of the global acreage [1]. Although male sterility is an unfavorable trait for individual plants, it plays critical roles in the utilization of heterosis by facilitating hybrid breeding [4]. Male sterility can be classified into cytoplasmic male sterility (CMS) and genic male sterility (GMS). CMS is controlled by organelles genes and can be restored by nuclear restorer gene(s). GMS is controlled by nuclear genes. In this condition, male sterility occurs under restrictive environmental conditions and fertility under permissive conditions [4]. To date, two recessive mutants *male sterility 1and male sterility 5* (*ms1* and *ms5*) and three dominant mutants *Male sterility 2, Male sterility 3, and Male sterility 4* (*Ms2*, *Ms3*, and *Ms4*) have been identified in wheat [5–9]. *Ms1* encodes a phospholipid-binding protein [10, 11]. *Ms2* was the first cloned dominant male GMS gene and has been widely used for wheat breeding [12, 13]. Known as a glycosylphosphatidylinositol-anchored lipid transfer protein, *Ms5* is required for normal pollen exine development [14].

Anther and pollen development have been widely studied in *Arabidopsis* [15], rice [16, 17], maize [2] and wheat [18]. Pollen development in those species involves similar key stages, which include microsporogenesis and male gametogenesis stages [2, 19, 20]. During the microsporogenesis stage, archesporial cells differentiate into microspore mother cells (MMCs), which finally undergo meiosis to generate microspores. During cytokinesis in plants, the parent cell divides into two daughter cells via physical insertion of a membranous cell plate [21]. The phragmoplast, a specialized cytoskeletal array, expands centrifugally during cytokinesis and directs Golgi-derived vesicles to form the developing cell plate. This process involves extensive protein secretion and membrane trafficking toward the plane of cell division [22]. During the male gametogenesis stage, the vacuolated microspore undergoes mitosis twice to produce a larger vegetative cell and a pair of sperm cells [23].

DNA methylation is a kind of epigenetic modification that occurs in plant genomes. Epigenetic modification of a single locus would result in heritable morphological variations without DNA sequence alterations [24, 25]. DNA methylation has been shown to participate in many plant development processes, including flower tissue development, pollen fertility, fruit ripening, and stress responses [26–30]. DNA methylation in plants occurs in symmetrical CG, CHG, and asymmetrical CHH (H represents A, T, or C) contexts [31]. CHG and CHH DNA methylation usually participate in heterochromatin formation and gene expression silencing, while the methylation sites in gene bodies are predominantly in the CG context [32, 33]. Male reproductive organs are more sensitive to damage from environmental change than vegetative organs [34]. There is increasing evidence that epigenetic regulation is essential for male sterility in plants. DNA demethylation was reported in vegetative and sperm cells, which reactivated transposable elements (TEs) and transposition in *Arabidopsis* [35]. In recent studies, changes were detected in the levels of DNA methylation in cotton (*Gossypium hirsutum*) anthers under high temperature (HT) in both HT-tolerant and HT-sensitive cotton cultivars [29, 30]. In rice, methylation in the putative promoter region of long-day-specific male-fertility-associated RNA (LDMAR) reduced its transcription level specifically under long-day conditions, which resulted in premature programmed cell death (PCD) in developing anthers, thus causing photoperiod-sensitive male sterility [36]. In the rice photoperiod- and thermo-sensitive genic male sterile (PTGMS) line PA64S, DNA methylation patterns were compared under sterile and fertile environments, and the results indicated that the hypermethylated *BIM2* gene might suppress downstream genes in the brassinosteroid signaling pathway and thus affect male fertility in PA64S [37].

Known as the prerequisite component for the heterosis breeding system, the male sterile line determines the hybrid yield and seed purity. Therefore, a deep understanding of wheat fertility and the mechanisms and gene networks that lead to male sterility are needed. To date, three wheat male sterile genes, *TaMs1*, *TaMs2*, and *TaMs5,* have been cloned; however, no studies on the cloning of temperature- or photo-sensitive male sterile genes have been reported in wheat thus far. In this study, transcriptome and reduced methylome sequencing were carried out for a temperature-sensitive genic male sterility (TGMS) BS366 and J411 (Jing411, a normal inbred line) under male sterile (12 °C with 12 h of daylight) and fertile conditions (20 °C with 12 h of daylight). Differentially expressed and methylated genes functioning in the cell division, carbohydrate and lipid metabolism pathways were identified. Our study provides novel insight into the role of DNA methylation in male sterility in wheat.

## Results

### Anther and pollen development was defective in BS366 under cold conditions

**Fig. 1:**
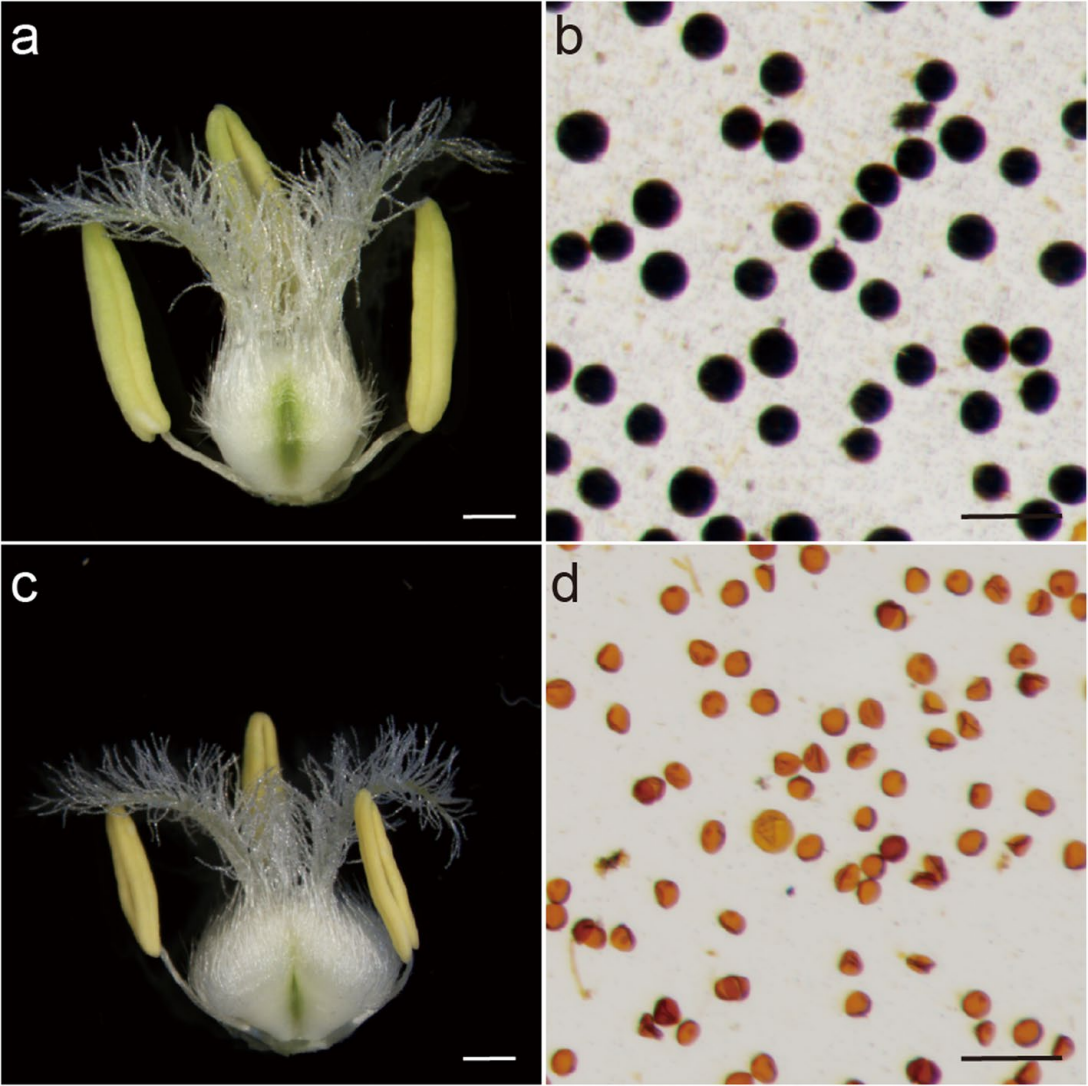
Phenotype comparison of BS366 anthers and pollen under cold (sterile) and control (fertile) conditions. **a** The mature anther of BS366 under control condition. **b** Pollen grains of BS366 under control condition stained with I_2_-KI. **c** The mature anther of BS366 under cold condition. **d** Pollen grains of BS366 under cold condition stained with I_2_-KI. Bars in **a** and **b**, 2 mm; bars in **c** and **d** 100 μm

**Fig. 2:**
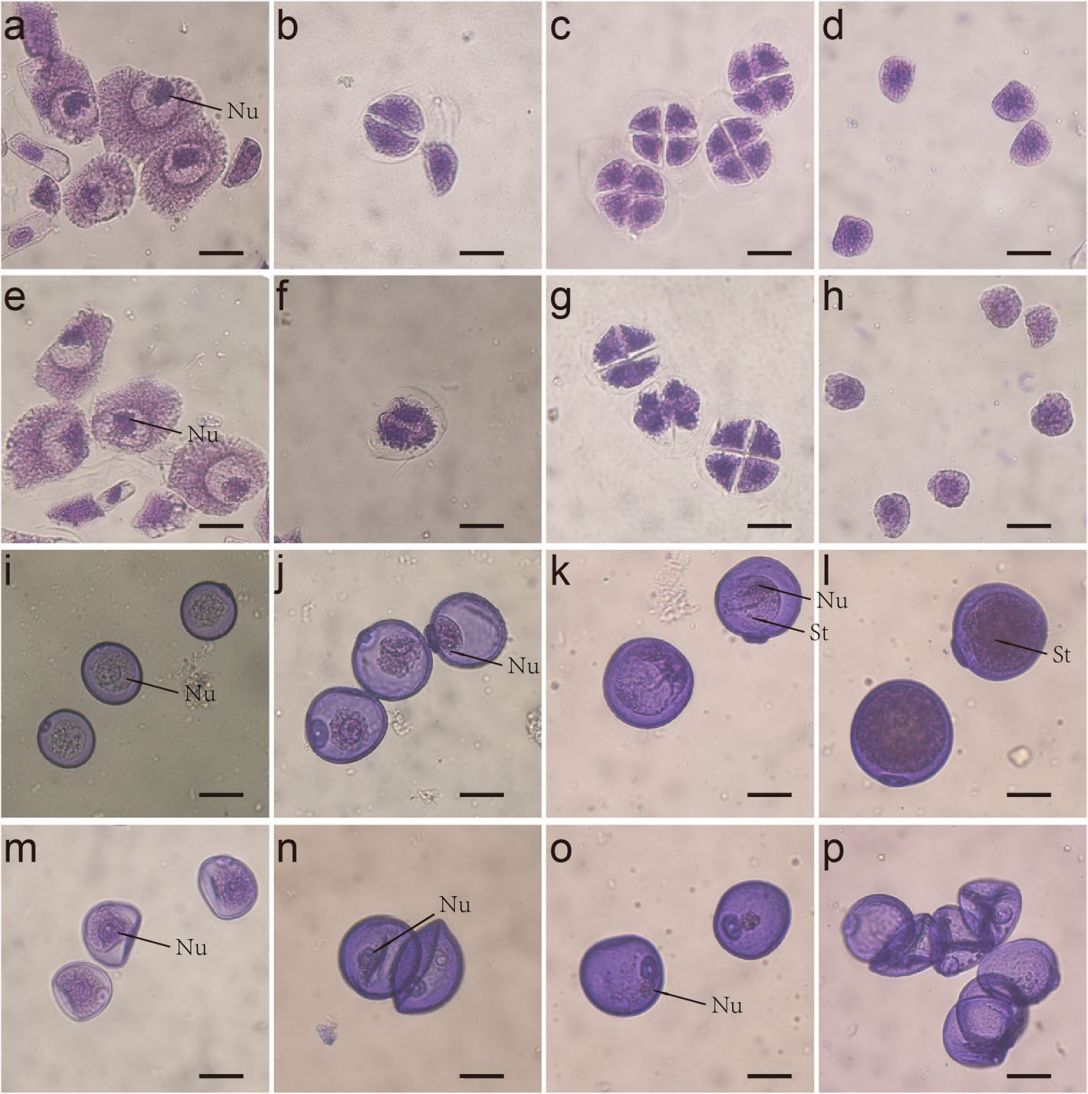
The microspores in fertile and sterile BS366. Micropores of fertile (**a**-**d**, **i**-**l**) and sterile BS366 (**e**-**h**, **m**-**p**). Meiotic interphase (**a**, **e**), meiotic dyad (**b**, **f**), meiotic tetrad (**c**, **g**), early uninucleate stage (**d**, **h**), middle uninucleate stage (**i**, **m**), vacuolated stage (**j**, **n**), binucleate stage (**k**, **o**) and mature pollen stage (**l**, **p**). Nu, nucleus; St, starch granules. Bars = 20 μm

Wheat TGMS line BS366 (Beijing Sterility 366) was selected from a natural mutant of doubled haploid lines (offspring of Jingnong8121/E8075–7) in the experimental fields in Beijing. Phenotypically, BS366 is normal at 20 °C but produces sterile pollen at 12 °C with 12 h of daylight. BS366 was used for phenotype and transcriptome analysis to study the temperature-sensitive male sterility in wheat. The fertile BS366 has normal anther and pollens (Fig. 1a and b). The sterile anthers are short and light yellow without pollens stained by I_2_-KI (Fig. 1c and d). The pollen and anther morphology of BS366 under cold and control conditions was examined during successive developmental stages. No difference is observed between the pollen mother cells (PMCs) of sterile and fertile BS366(Fig. 2a and e). Compared with dyad and tetrad in control (Fig. 2b and c), some dyads in the cold-treated BS366 lacked a smooth cell plate in telophase I (Fig. 2f). A more severe defect was observed in the cell plate (Fig. 2g), which resulted in an abnormal tetrad and thus unable to release uninucleate pollen grains. Compared with pollens at early (Fig. 2d and h) and middle uninucleate stages (Fig. 2i and m) under control conditions, the pollen grains under cold-treated BS366 shrank at the vacuolated stage (Fig. 2j and n). As the starch accumulated inside the microspore, the vacuole gradually diminished in the fertile pollens (Fig. 2k and l). In the sterile pollen, no starch accumulated, and a smaller nucleus without generative and vegetative cells was observed (Fig. 2o). The sterile pollen was vacuolated (Fig. 2p).

**Fig. 3:**
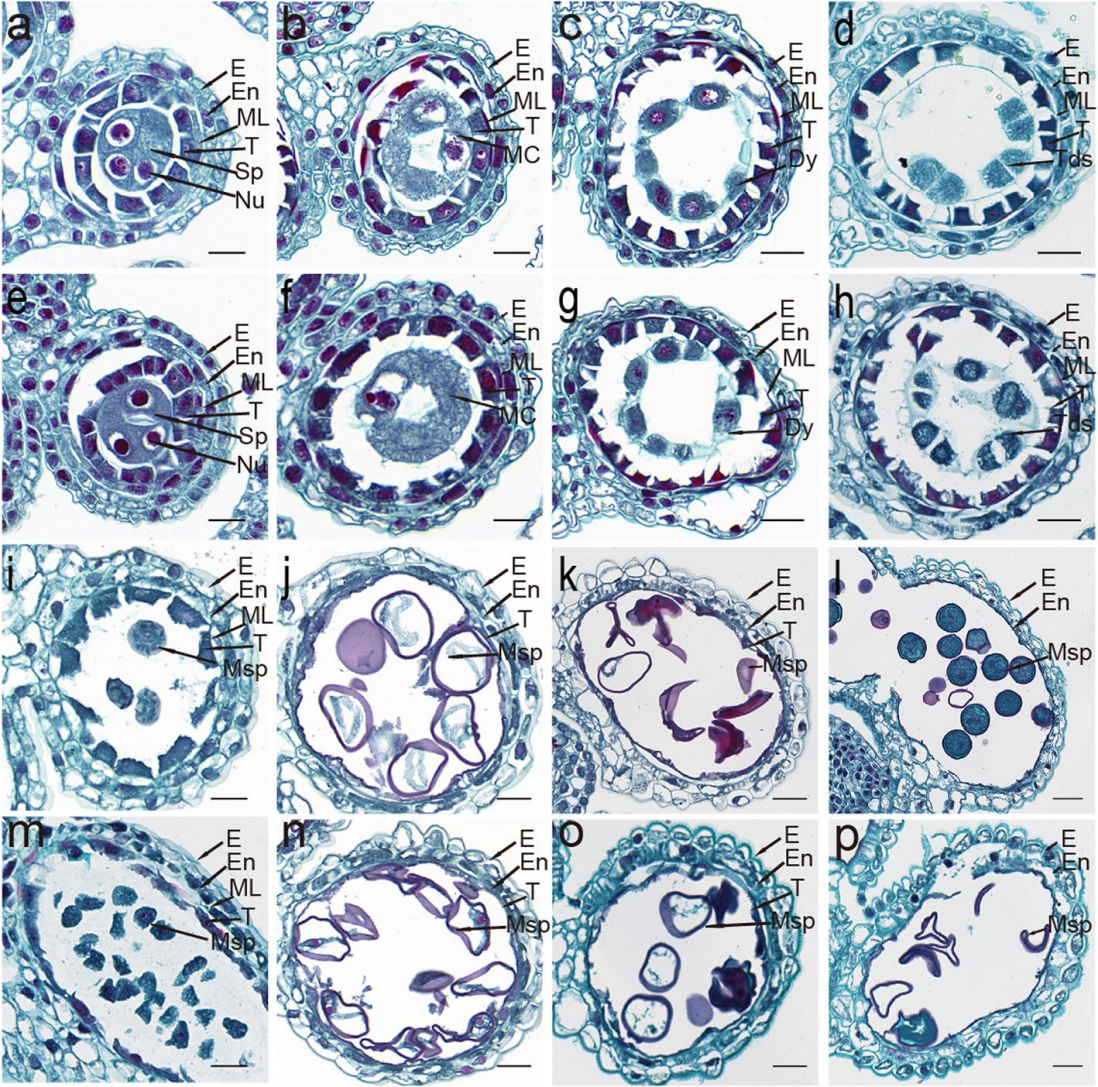
Transverse sections of BS366 anthers from different developmental stages under cold and control conditions. Anthers in fertile (**a**-**d**, **i**-**l**) and sterile BS366 (**e**-**h**, **m**-**p**). Precallose stage (**a**, **e**), meiotic stage (**b**, **f**), dyad stage (**c**, **g**), tetrad stage (**d**, **h**), early uninucleate stage (**i**, **m**), vacuolated stage (**j**, **n**), binucleate stage (**k**, **o**), and trinucleate stage. (**l**, **p**). Dy, dyad cell; E, epidermis; En, endothecium; ML, middle layer; Msp, microspores; T, tapetum; Tds, tetrads; Nu, nucleus. Bars = 50 μm

To characterize the histological differences between fertile and sterile anthers, transverse sections of anthers embedded in paraffin and stained with safranin O-fast green were observed. The sporogenous cells, epidermis, endothecium, middle layer, and tapetum showed no difference between anthers from sterile and fertile BS366. No abnormal structures were observed at the precallose (Fig. 3a and e) or meiotic stage (Fig. 3b and f). Compared with the fertile anther (Fig. 3c and d), a serious defect of the cell plate in the dyad and tetrad was observed (Fig. 3g and h). At the vacuolated stage, round and vacuolated microspores were observed (Fig. 3i and m). Compared with fertile BS366 (Fig. 3j), sterile microspores were swollen and became less vacuolated and collapsed (Fig. 3n). The fertile pollen started to accumulate starch grains at the binucleate stage (Fig. 3k), while few starch grains were observed in the sterile pollen (Fig. 3o). At the mature stage, the fertile anther locule was full of mature pollen grains, and anther dehiscence occurred, leaving only the epidermis and endothecium layers (Fig. 3l). However, the sterile middle layer and endothecium became abnormally expanded and thicker, and the microspores had an irregular appearance. In particular, the endothecium near the connective tissues expanded, and the pollen disintegrated into debris (Fig. 3p).

### Profiling the transcriptome of BS366 and J411 under different conditions

To eliminate the cold responses that are not related to male sterility, a normal inbred line, Jing411 (J411) is used as control. Young spikes of BS366 and J411 from cold and control conditions of different anther development stages were pooled together for transcriptome sequencing. Transcriptome sequence data for all samples can be found in the National Genomics Data Center (https://bigd.big.ac.cn/) under accession number CRA003366. After filtering and quality control of the raw reads, a total of 910,342,786 clean reads were generated, with an average of 96,761,244 reads per sample. Finally, 85.07% (774,089,948/910,342,786) of the reads were mapped to the reference genome(IWGSC RefSeq v1.1), of which 86.16% (666,921,316) were uniquely mapped (Table 1). The correlation between biological replicates varied between 0.87–0.94 for BS366 and J411 under fertile and sterile conditions (Additional file 1: Fig. S1), which indicated good replication of those samples.

**Table 1:** Mapping statistics for all the samples in this study

Sample	Total Reads	Total Mapped reads	Mapping Rate	Unique Mapped reads	Unique mapping rate
BS366_con_1	113,245,958	98,668,138	87.13%	82,899,792	73.20%
BS366_con_2	112,097,060	95,441,688	85.14%	82,398,866	73.51%
J411_con_1	116,496,542	94,178,836	80.84%	78,483,052	67.37%
J411_con_2	114,294,642	98,351,910	86.05%	85,569,280	74.87%
BS366_col_1	118,214,624	100,855,918	85.32%	86,617,232	73.27%
BS366_col_2	125,833,982	106,080,724	84.30%	91,159,560	72.44%
J411_col_1	104,066,440	89,588,230	86.09%	79,396,020	76.29%
J411_col_2	106,093,538	90,924,504	85.70%	80,397,514	75.78%

### Differentially expressed genes and functional analysis

**Fig. 4:**
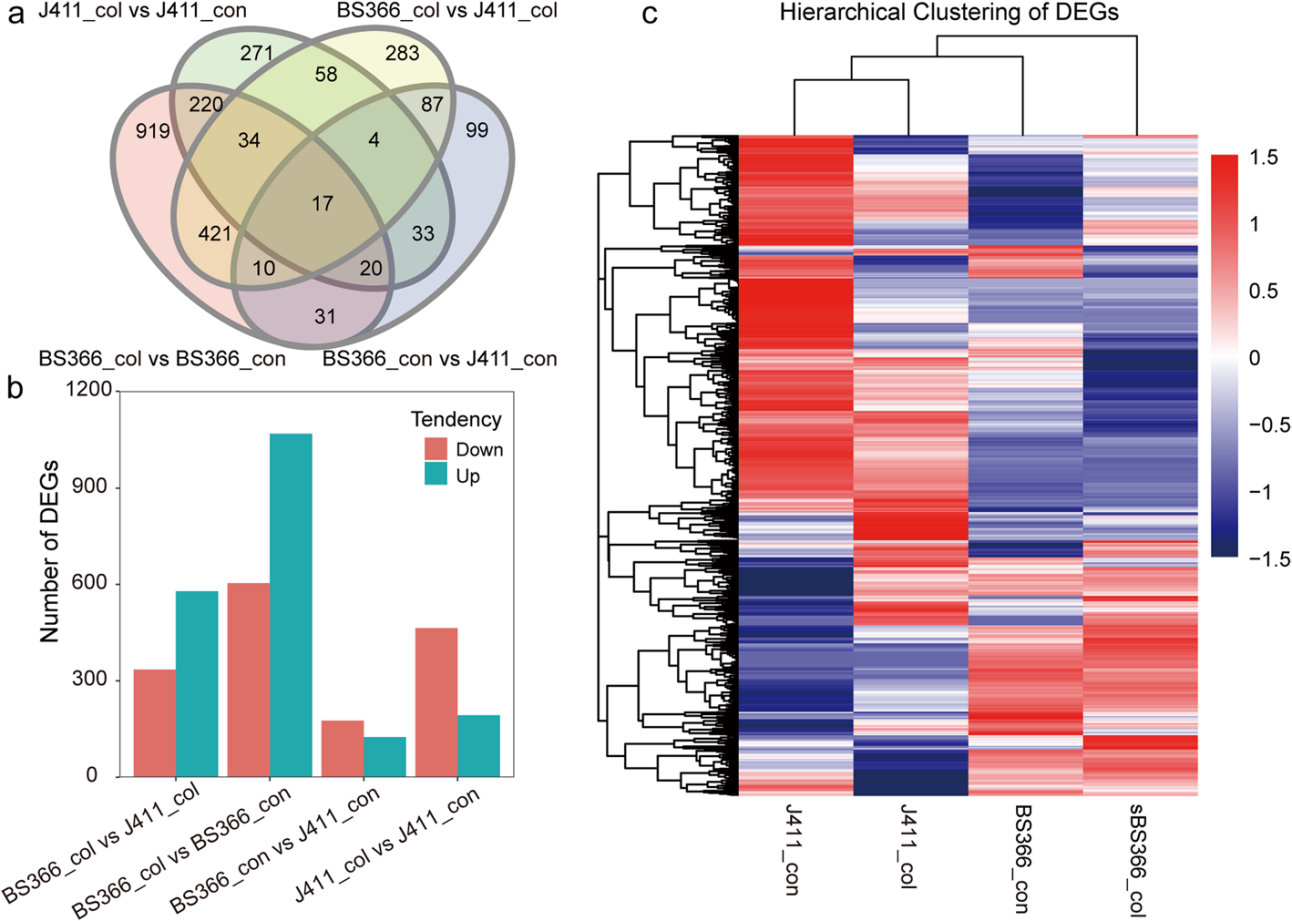
All differentially expressed genes in the transcriptome sequencing data. **a** Venn diagram of all the differentially expressed genes in each comparison. The col. and con indicate cold and control conditions. **b** Number of differentially expressed genes that were up- or down-regulated in each comparison. Up means upregulated under the sterile condition in comparison to the different conditions or in BS366 in comparisons between different samples under the same conditions. **c** Hierarchical cluster analysis of all differentially expressed genes. The color key represents the normalized log_2_ transformed expression of genes, with genes with expression shown in red and those with low expression shown in blue

To characterize the expression changes of putative candidate genes involved in male sterility under cold conditions in BS366 spikes, the numbers of differentially expressed genes (DEGs) were calculated. Fragments per kb of exon model per million mapped reads (FPKM) was used to estimate the transcript expression levels in all samples. In BS366, 7879 genes were expressed, among which 4438 genes were expressed under normal and 6879 genes were expressed under cold conditions. In total, 7415 genes were expressed in J411, with 4573 genes expressed under normal and 6438 genes expressed under cold conditions. The genes expressed in BS366 and J411 under normal/cold conditions were approximately the same. A total of 2507 genes were identified as differentially expressed in this study, with 1672 in BS366 and 657 in J411 between control and cold conditions (Fig. 4a and c). There were 301 DEGs under the control condition and 914 DEGs under the cold condition between BS366 and J411. As shown in Fig. 4a, there are 1381 genes exclusively differentially expressed between cold and control conditions in BS366 compared with that in J411.

To illustrate the alteration of the transcriptome accompanying the change in temperature, the DEGs were classified as up- or down-regulated under the cold conditions and/or BS366 according to their expression levels. Finally, 1068 up-regulated and 604 down-regulated genes were found in sterile BS366 spikes. In J411, 193 up-regulated and 464 down-regulated genes were identified under sterile conditions compared with the control. Compared with J411, there were 125 up-regulated and 176 down-regulated genes in BS366 under control conditions and 579 up-regulated and 335 down-regulated genes in BS366 under cold conditions (Fig. 4b). These results revealed a more rigorous transcriptome change in BS366 than J411 between cold and control conditions.

The exclusive 1381 DEGs in BS366 between cold and control conditions may contribute to male sterility in BS366_col. Thus, Gene Ontology (GO) analysis was performed for those BS366-specific DEGs. Among the top 20 GO terms of molecular function, structural molecule activity and binding activities, such as binding, protein binding, nucleic acid binding, RNA binding, mRNA binding, heterocyclic compound binding, organic cyclic compound binding, and DNA binding were also significantly represented. Molecular activities like structural constituent of cytoskeleton, microtubule motor activity, microtubule binding, tubulin binding were also significantly represented (Additional file 2: Table S1).

In the biological process category, DNA conformation change, nucleosome organization, and chromosome organization, which have been reported to be common for male sterility in other plants were significantly represented. Other biological processes, including sister chromatid segregation, microtubule-based process, mitotic sister chromatid segregation, mitotic cell cycle process, cell cycle process, sister chromatid cohesion, mitotic nuclear division, cell cycle, microtubule-based movement, and mitotic cell cycle were also significantly represented. Biological processes involved in epigenetic regulation such as regulation of gene expression, epigenetic, negative regulation of gene expression, macromolecule methylation, DNA methylation, methylation, DNA methylation or demethylation, DNA methylation on cytosine, and histone lysine methylation were significantly represented in the BS366-specific DEGs (Additional file 2: Table S1). Compared with biological processes enriched in BS366- specific DEGs between cold and control conditions, there were only seven significantly (corrected *p*-value<0.05) enriched biological processes in J411, including phosphorylation, phosphorus metabolic process, phosphate-containing compound metabolic process, protein phosphorylation, ATP synthesis coupled electron transport, oxidative phosphorylation, respiratory electron transport chain (Additional file 2: Table S1). All BS366-specific DEG enrichment processes indicated that it was a comprehensive dynamic molecular network responsible for male sterility in BS366 cells.

### Coexpression module analysis of all differentially expressed genes

**Fig. 5:**
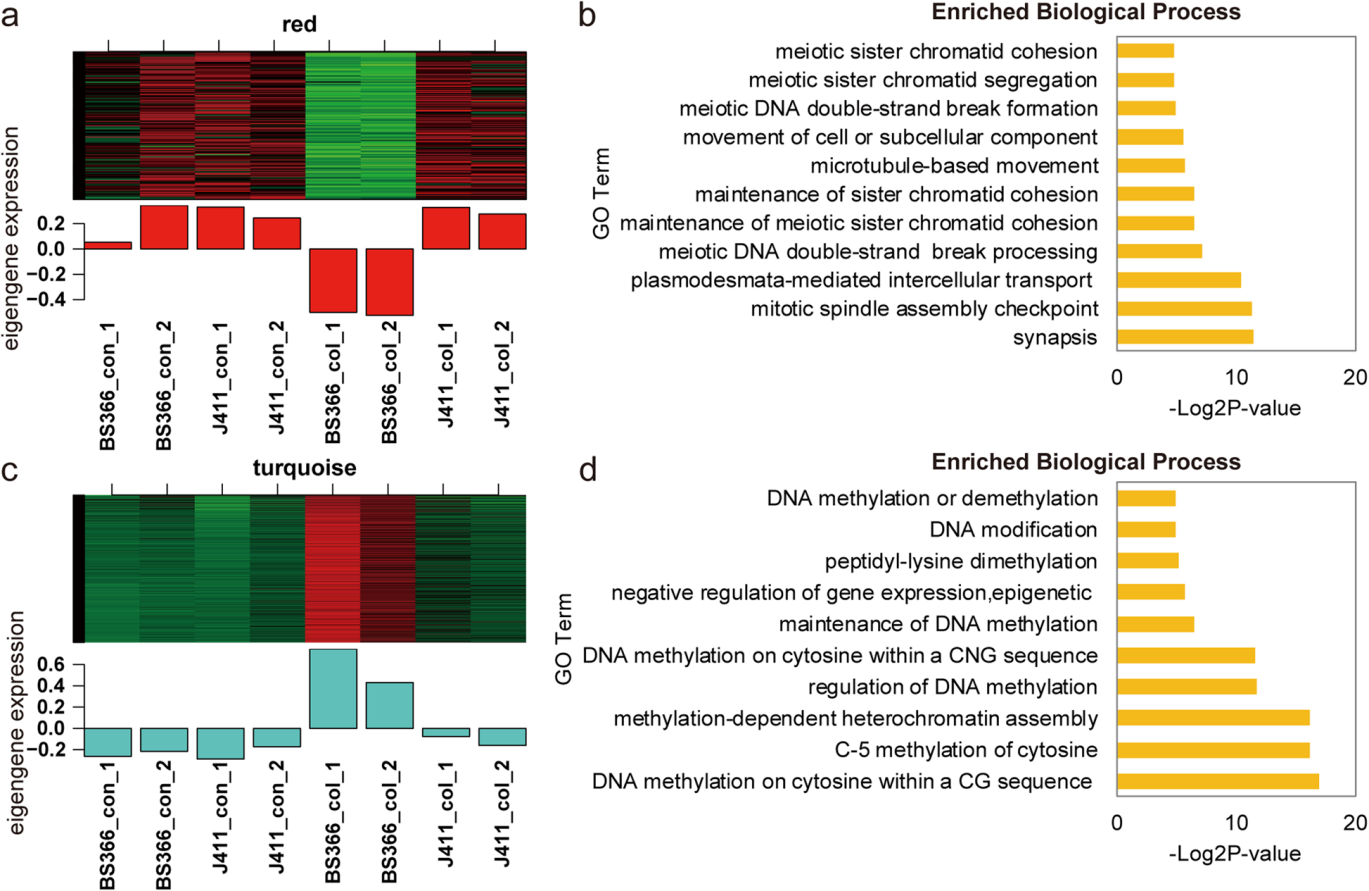
The expression patterns and functional analysis of genes in the coexpression modules. **a** Expression pattern of genes in the red module. **b** GO analysis of genes in the red module. **c** Expression pattern of genes in the turquoise module. **d** GO analysis of genes in the red module. In the heatmap, red indicates up-regulated genes, black indicates neutral genes, and green indicates down-regulated genes

All 2507 DEGs between different conditions and/or materials were used to construct coexpression modules. Finally, 2329 genes were assigned to 12 expression modules, for which the gene number ranged from 35 (tan) to 1022 (turquoise) (Additional file 1: Figs. S2 and S3). As shown in Fig. 5, most of the DEGs (1023, 43.88%) were assigned to the turquoise module, in which all the genes exhibited lower expression levels in BS366_con, J411_con, and J411_col but a higher expression level in BS366_col. Compared with genes in the turquoise module, 142 genes in the red module exhibited an opposite expression pattern. Only genes in BS366_col were expressed at high levels compared with the other samples (Fig. 5a and c).

To gain an overall understanding of the genes assigned to the two opposite modules, GO analysis was implemented for both modules. In the red module, genes were enriched in biological processes related to meiotic sister chromatid segregation and cohesion, mRNA cleavage involved in gene silencing, and microtubule-based movement (Fig. 5b and Additional file 2: Table S2). In the turquoise module, genes were mainly enriched in processes related to DNA methylation and histone modification, microtubule-related processes and movement. Genes assigned to the molecular function category were significantly enriched in methyl-CpG binding, histone-binding, DNA (cytosine-5)-methyltransferase activity, 5-methyltetrahydropteroyltriglutamate-homocysteine S-methyltransferase activity, and histone methyltransferase activity (H3-K4 specific). In the cellular component category, genes were significantly enriched in the MCM complex, cell wall, tubulin complex, microtubule, and mitotic spindle. In the molecular function category, those genes were enriched in microtubule motor activity, DNA (cytosine-5)-methyltransferase activity, microtubule binding, histone methyltransferase activity (H3-K4 specific), calcium-transporting ATPase activity, 1,3-beta-D-glucan synthase activity, methyl-CpNpG binding, methyl-CpNpN binding, methyl-CpG binding, methylated histone binding, and methylation-dependent protein binding (Fig. 5d and Additional file 2: Table S3).

Similar to DNA methylation, many histone constitution- and modification-related genes were found in the turquoise module. A total of 68 histone constitution genes (such as histone H1, histone H2A, histone H2B, and histone H3) showed expression peaks in BS366_col (Additional file 1: Fig. S4a). Similarly, eight genes involved in histone modification also showed higher expression levels in BS366_col than in other samples (Additional file 1: Fig. S4b). These genes encoded proteins such as histone-lysine N-methyltransferase ATXR5, histone-lysine N-methyltransferase, putative histone-lysine N-methyltransferase ATXR3, histone-lysine N-methyltransferase H3 lysine-9 specific SUVH5, and histone deacetylase HDAC2. Seven genes encoding proteins homologous to DNA (cytosine-5)-methyltransferase 1 (DRM1) or DNA (cytosine-5)-methyltransferase 1B in *Aegilops tauschii* or *Triticum urartu* showed higher expression levels in BS366_col compared with other samples (Additional file 1: Fig. S4c). Based on the above results, we could conclude that induced expression of DNA methylation-related genes was likely to cause the higher methylation level in BS366_col; the higher expression of histone constitution genes in BS366_col might allow anthers to maintain DNA stability in BS366_col. These results indicated that epigenetic modifications, particularly DNA methylation and histone modifications, were involved in anther development under low-temperature conditions.

### Expression validation of differentially expressed genes in transcriptome data

Finally, 19 DEGs in BS366 between control and cold conditions were selected for validation using real-time qRT-PCR. The Pearson’s correlation coefficient between data generated from the two platforms was very high (*R*^2^ = 0.93), indicating that RNA-seq analysis generated dependable data (Additional file 1: Fig. S5).

### Genome-wide DNA methylation in BS366 and J411 spikes under two conditions

The exclusive DEGs involved in DNA methylation and histone modification suggested that DNA methylation might be involved in the male sterility of BS366 spikes. To delineate the role of DNA methylation in pollen sterility, MethylRAD analysis [38], a cost-efficient DNA methylation profiling method, was used to characterize the cytosine methylation patterns of spikes. Eight samples for BS366 and J411 under both cold and control conditions were sampled and prepared for the MethylRAD sequencing library. In total, 1,089,514,960 raw reads were obtained, of which 364,356,240 (33.44%) enzyme-digested reads were identified. There were 359,562,630 enzyme-digested reads mapped to the reference wheat genome (IWGSC RefSeq v1.0), of which 31,327,447 reads were uniquely mapped (Table 2). MethylRAD sequence data for all samples can be found in the National Genomics Data Center (https://bigd.big.ac.cn/) under accession number CRA003366. In this analysis, only the uniquely mapped reads were retained to measure the methylation level of loci.

**Table 2:** The mapping statistics for all the reads from all the MethylRAD libraries

Samples	Raw Reads	Enzyme Reads	Ratio	Unique Mapped Reads	Multiple Mapped Reads	Unique Mapping ratio	Multiple Mapping ratio
BS366_col_1	138,774,718	49,583,815	35.73%	4,321,361	44,579,071	8.72%	89.91%
BS366_col_2	138,774,718	45,195,153	32.57%	3,824,533	40,805,978	8.46%	90.29%
BS366_con_1	138,774,718	49,388,164	35.59%	4,244,368	44,521,958	8.59%	90.15%
BS366_con_2	138,774,718	48,491,659	34.94%	4,227,335	43,659,713	8.72%	90.04%
J411_col_1	133,604,022	43,190,796	32.33%	3,688,005	38,865,858	8.54%	89.99%
J411_col_2	133,604,022	44,081,539	32.99%	3,729,612	39,787,159	8.46%	90.26%
J411_con_1	133,604,022	42,870,752	32.09%	3,721,482	38,571,647	8.68%	89.97%
J411_con_2	133,604,022	41,554,362	31.10%	3,570,751	37,443,799	8.59%	90.11%
All	1,089,514,960	364,356,240	33.44%	31,327,447	328,235,183	8.60%	90.09%

**Fig. 6:**
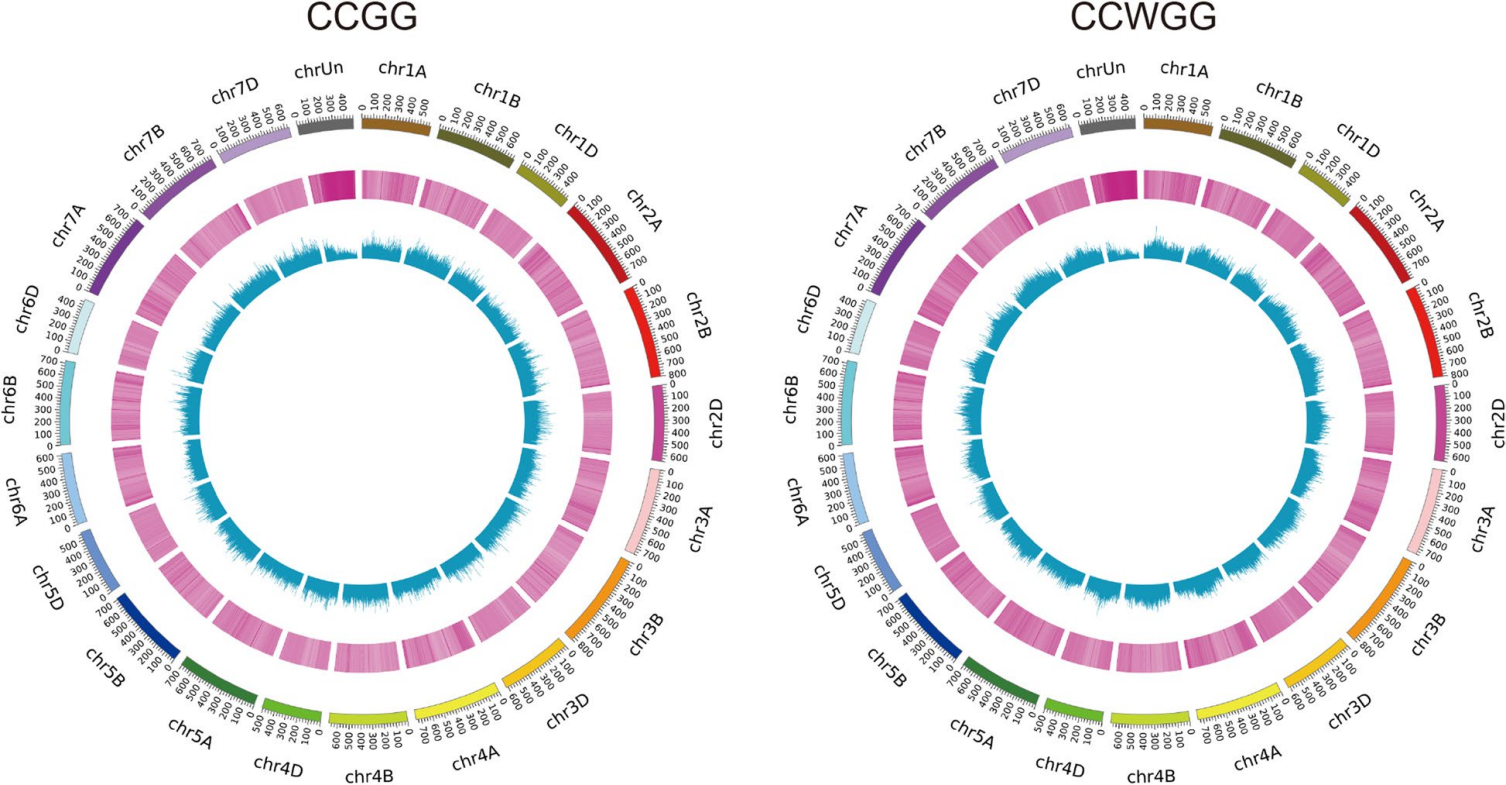
Genome distribution of the methylated sites in this study. From the inside to the outside is shown the histogram, heatmap, and chromosome location of the methylated sites

**Fig. 7:**
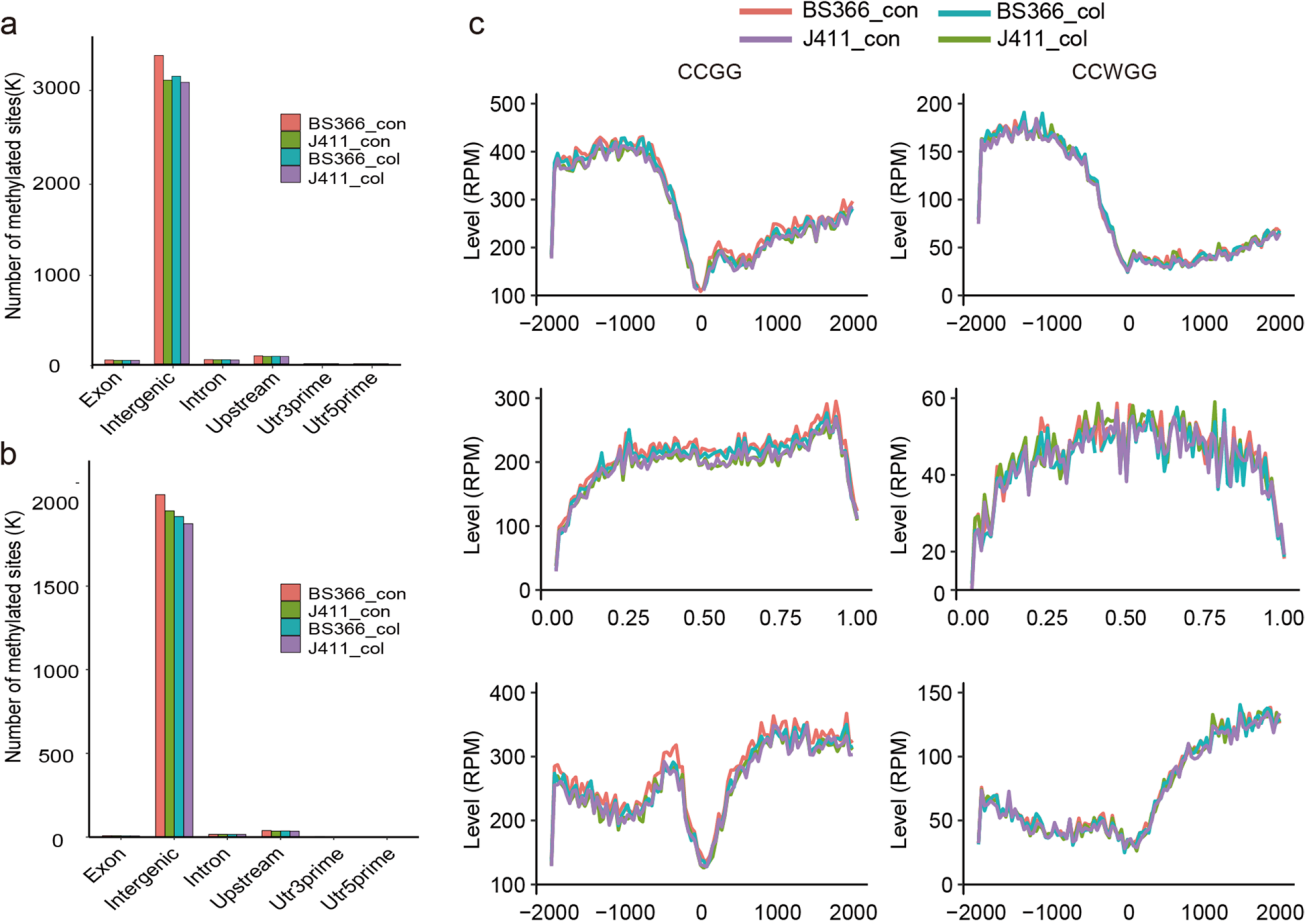
Genome distribution of the methylation patterns in this study. **a** Distribution of the CCGG sites on the genome elements for each sample. **b** Distribution of the CCWGG sites on the genome elements for each sample. **C** Analysis of the CCGG and CCWGG levels in the gene regions, including promoters (− 2000 bp), gene bodies and downstream regions (+ 2000 bp), in BS366 and J411 under cold and control conditions. Upper panel, methylation level in the TSS region; middle panel, methylation level in the gene body region; lower panel, methylation level in the TTS region. TSS, transcription start site; TTS, transcription termination sites

Using the MethylRAD method, we analyzed DNA methylation at CG and CHG (H = T or A) sites throughout the genome. As shown in Fig. 6 and Additional file 2: Table S4, there were 3,238,375 CCGG and 1,936,460 CCWGG sites in BS366_col and 3,473,022 CCGG and 2,069,354 CCWGG sites in BS366_con. In J411, there were 3,167,380 and 1,893,560 CCGG and CCWGG sites in J411_col and 3,191,535 and 1,971,193 methylated CCGG and CCWGG sites in J411_con, respectively. The methylation profiles within genes, including promoters, exons, and introns, were analyzed for four samples. Clearly, the CCGG context showed a higher methylation level than the CCWGG context in the gene region. However, the distribution patterns of methylated sites at different elements of genomes were similar for the CCGG and CCWGG sites. The CCGG and CCWGG sites in the intergenic regions were easily methylated, followed by the upstream 2000-bp region of the transcription start site (TSS2000), intron, exon, Utr3prime, and Utr5prime regions (Fig. 7a and b). According to the MethylRAD method, we compared the methylation profiles of sites in the up- and down-stream 2000 bp of the TSS, TSS and gene body. We found that the methylation context was higher for CCGG than for CCWGG for the above three characteristics. The methylation level was higher in the upstream 2000 bp than in the gene body and TTS region for both the CCGG and CCWGG contexts. The methylation levels were slightly higher for the TSS region than for the gene body region in both the CCGG and CCWGG contexts (Fig. 7c).

### Differentially methylated sites and functional analysis

**Fig. 8:**
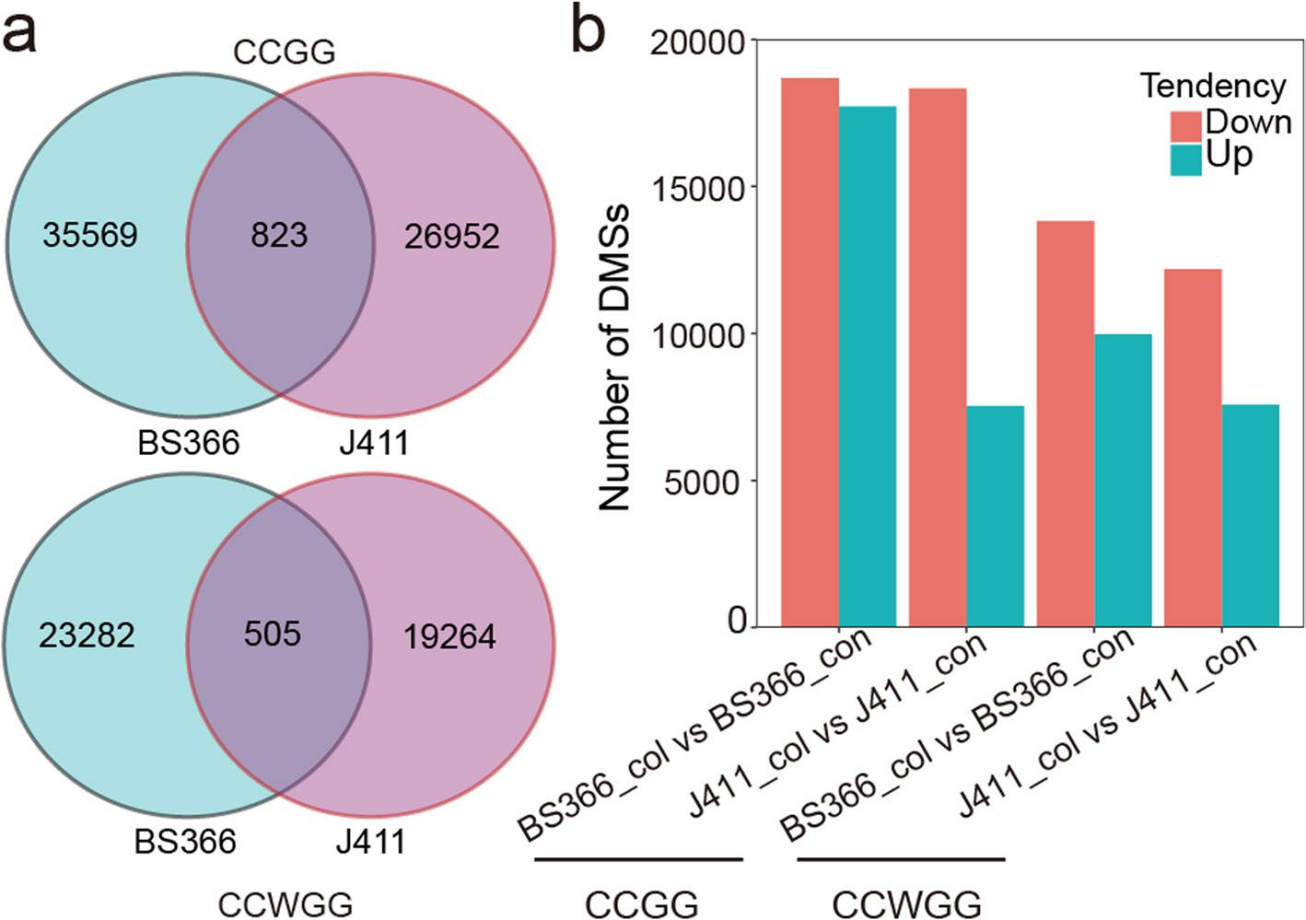
Differentially methylated sites in BS366 and J411 between cold and control conditions. **a** Venn diagram analysis of the differentially methylated CCGG and CCWGG sites in BS366 and J411 under different conditions. **b** All the differentially methylated sites were classified as hyper and hypomethylated sites in BS366 spikes or under cold conditions. Up indicates hypermethylation in BS366 spikes or under cold conditions; down indicates hypomethylation in BS366 spikes or under cold conditions

**Fig. 9:**
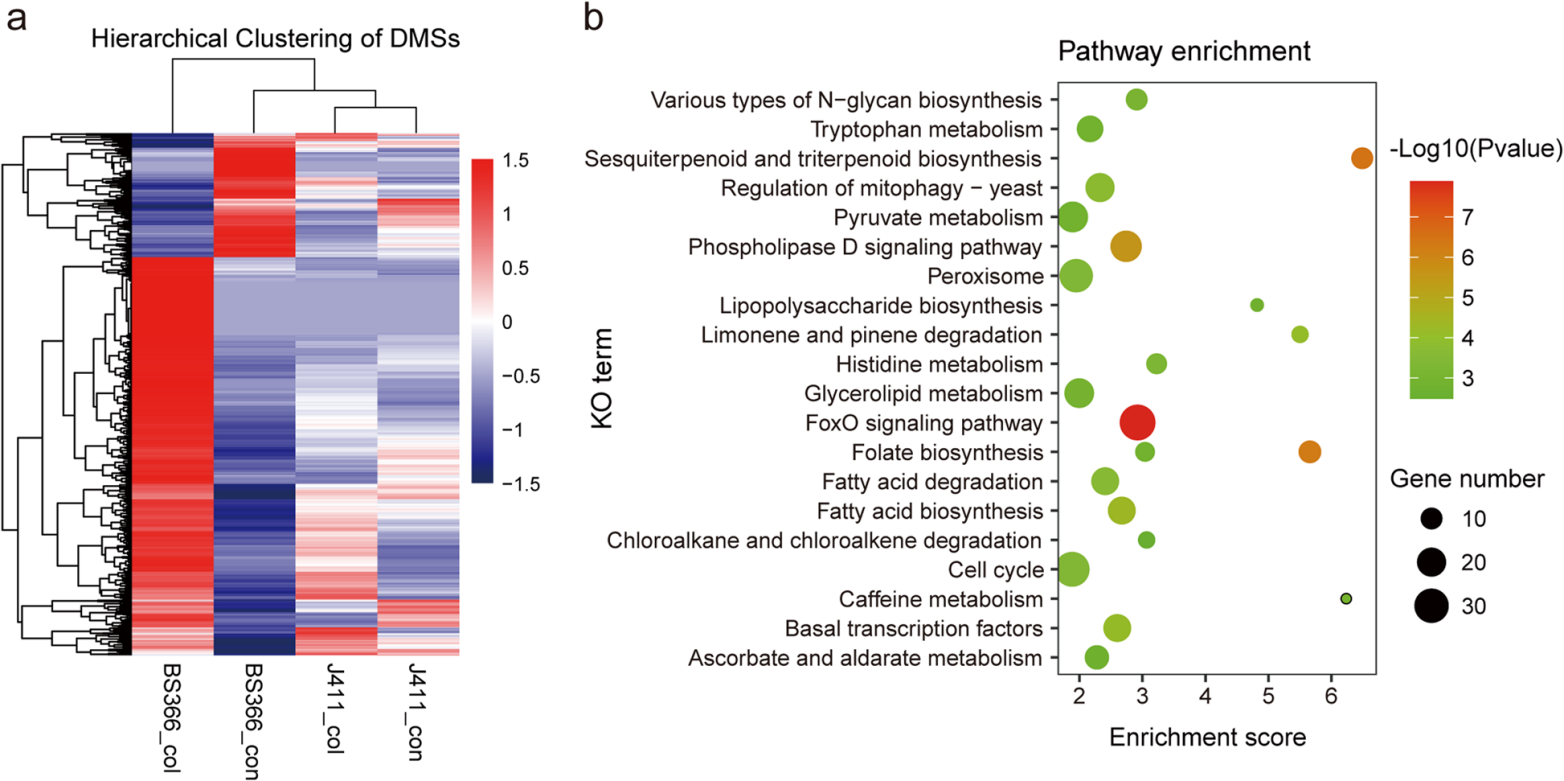
Exclusive DMSs in BS366 between cold and control conditions. **a** Expression heatmap of the exclusive DMSs in BS366 spikes under cold and control conditions. **b** Kyoto Encyclopedia of Genes and Genomes (KEGG) analysis of the exclusive DMSs in BS366 between cold and control conditions. The color scale indicates the methylation levels: red indicates high methylation levels and navy indicates low methylation levels

To further identify the function of methylation changes among gene features in response to cold stress, the differentially methylated sites (DMSs) in BS366 and J411 between different conditions were characterized using the criteria of |log 2FC| > 1 and a *P* value < 0.05. In total, 36,392 CCGG and 23,787 CCWGG loci were detected as differentially methylated in BS366 between cold and control conditions. More DMSs were detected in BS366 than in J411 for both CCGG and CCWGG sites between the two conditions (Fig. 8a). The DMSs were further divided into hypermethylated and hypomethylated sites. More DMSs were detected in BS366 between cold and control conditions than in J411 for both hypermethylated and hypomethylated sites. There were 17,715 and 9966 hypermethylated CCGG and CCWGG sites in BS366_col and 18,677 CCGG and 13,821 hypomethylated CCWGG sites in BS366_col. There were 9448 CCGG and 7528 CCWGG sites hypermethylated in J411_col; there were 18,327 CCGG and 12,187 CCWGG sites hypomethylated in J411_col (Fig. 8b). We further inspected the DMS distribution in the genome elements and found that the majority of the DMSs were located in the intergenic region (92.01–97.39%), followed by the exon, intron, upstream, utr3prime, and utr5prime regions for CCGG DMSs; the CCWGG DMSs were mainly located in the intergenic region upstream, intron, and exon regions (Additional file 1: Fig. S6). To explore the pathways important for male sterility in the BS366 response to cold conditions, DMSs specific for BS366 were annotated to the reference pathways in the KEGG database. In this analysis, 1130 CCGG and 633 CCWGG DMSs assigned to 1591 genes were retained (Fig. 9a). KEGG enrichment analysis revealed pathways, including the phospholipase D signaling pathway, fatty acid biosynthesis, fatty acid degradation, and peroxisome, were significantly enriched (Fig. 9b). All these results indicated that pathways involved in fatty acid metabolism, phenylpropanoid biosynthesis, and the phospholipase D signaling pathway might be regulated by DNA methylation to participate in male sterility in BS366 cells under cold conditions. In the GO analysis of the DMSs specific for BS366, biological processes including carbohydrate transport, oxylipin biosynthetic process, positive regulation of transcription from RNA polymerase II promoter, and lipid transport were also significantly represented (Additional file 2: Table S5).

### Genes differentially expressed and methylated under different conditions in BS366 spikes

**Fig. 10:**
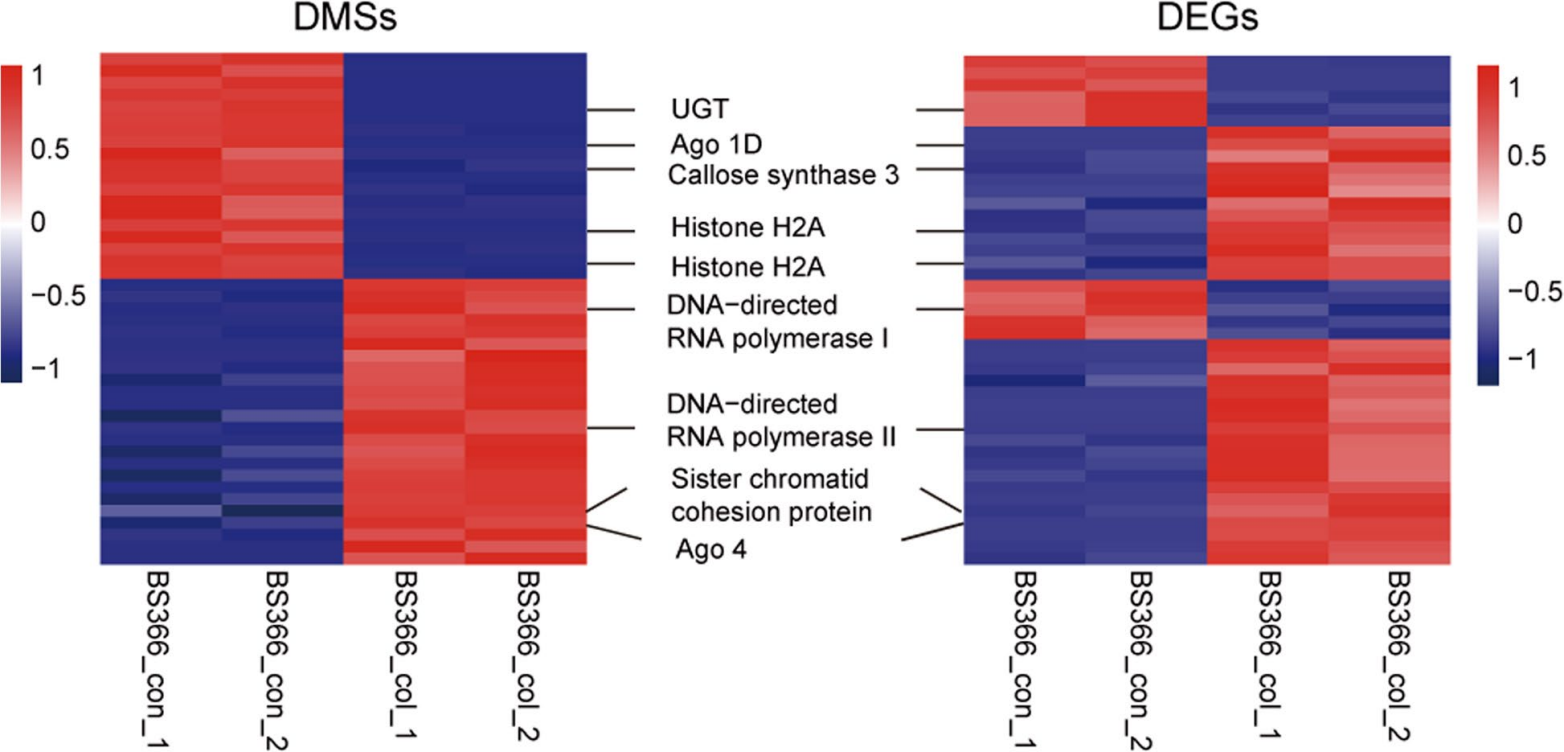
The expression and methylation level of genes from the integrated analysis of the transcriptome and methylation. Heatmap of the methylation level of DMSs in BS366 spikes (left). Heatmap of the expression level of DEGs in BS366 spikes (right). Red indicates hypermethylated or upregulated, and blue indicates means hypomethylated or downregulated

The DMSs in the methylome and DEGs in the transcriptome sequencing were integrated to investigate the role of DNA methylation in gene expression. Genes that were differentially expressed and methylated in BS366 cells under different conditions were identified. Finally, 49 DMSs in the methylome sequencing located in 45 DEGs in the transcriptome sequencing were identified. These genes were classified into four categories: 1) eight genes with lower methylation and expression levels; 2) 15 genes with lower methylation levels but higher expression levels; 3) five genes with higher methylation levels but lower expression levels; and 4) 21 genes with higher expression and methylation levels in BS366 under cold conditions (Fig. 10). Among those sites, 13 were located in the upstream region, eight were located in the intron region, and 36 were located in the exon region.

For instance, a gene encoding the AGO1d protein (TraesCS7A02G557400) was expressed at higher levels but hypomethylated in BS366_col in comparison to BS366_con. A gene encoding AGO4, a partial protein (TraesCS1A02G445500), was expressed and methylated at higher levels in BS366_col than in BS366_con. A homologous gene (TraesCS1B02G179900) of *DNA-directed RNA polymerase I subunit 2* in *Brachypodium distachyon* and a homologous gene (TraesCS6D02G113900) of *DNA-directed RNA polymerase II subunit RPB1-B* in *Aegilops tauschii* were hypermethylated in BS366_col compared with BS366_con. However, the methylation tendency of these two genes differed, with the former down-regulated and the latter up-regulated in BS366_col. A homologous gene (TraesCS7D02G206700) of *the chromatin structure-remodeling complex subunit snf21* in *Aegilops tauschii* was up-regulated but hypomethylated in BS366_col. The gene encoding sister chromatin cohesion protein PDS5-like protein B (TraesCS7A02G215200) was up-regulated and hypermethylated in BS366_col. Two genes encoding histone proteins, one homologous to the putative histone H2AXb in *Aegilops tauschii* (TraesCS5A02G098300) and H2A3 protein (TraesCS1B02G048900), were up-regulated but hypomethylated in BS366_col. One gene encoding ribosomal protein L19 (TraesCS2D02G092800) was up-regulated and hypermethylated in BS366_col (Fig. 10).

### Pyrosequencing validation of the differentially methylated sites

**Fig. 11:**
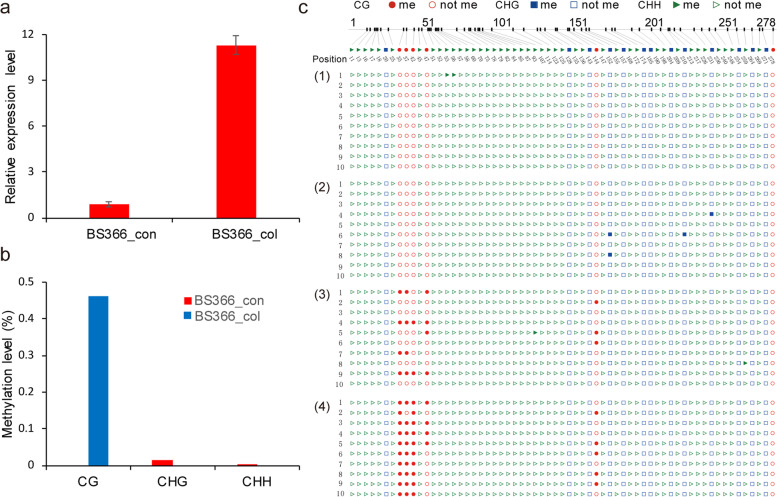
Expression and methylation level validation of TraesCS7A02G557400 (AGO1d) in BS366 spikes under cold and control conditions. **a** Expression level of BS366 under cold (BS366_col) and control (BS366_con) conditions. **b** Methylation level of the *AGO1d* gene in BS366 spikes under cold and control conditions. **c** Bisulfite sequencing of the differentially methylated sites in the *AGO1d* gene in cold (1, 2) and control (3, 4) condtions. The cycle, triangle, and rectangle represent the CG, CHG and CHH contexts. The filled red, green and blue cycles represent methylated cytosine, and the unfilled cycle, triangle and rectangle represent the unmethylated cytosine sites

**Fig. 12:**
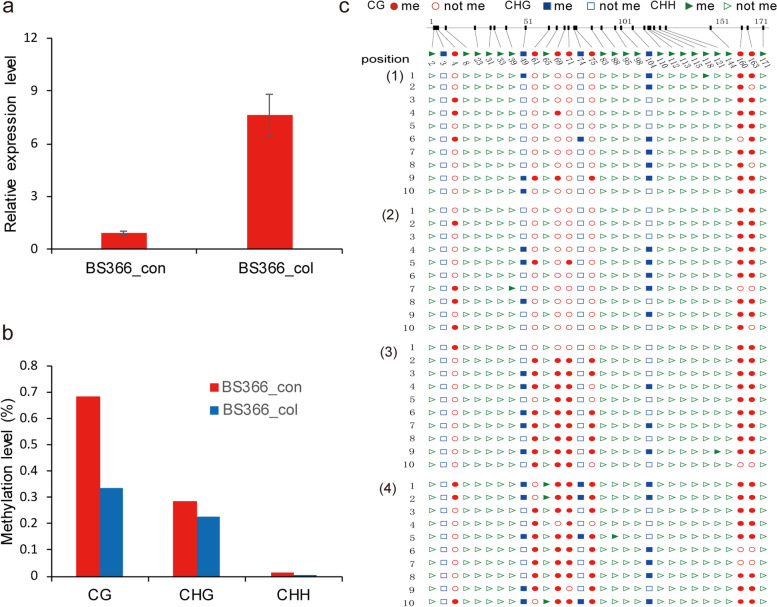
Expression and methylation level validation of TraesCS1B02G048900 (H2A3) in BS366 between cold and control conditions. **a** Expression level of BS366 under cold (BS366_col) and control (BS366_con) conditions. **b** Methylation level of TraesCS1B02G048900 in BS366 under cold and control conditions. **c** Bisulfite sequencing of differentially methylated sites in the TraesCS1B02G048900 gene in cold (1, 2) and control (3, 4) condtions. The cycle, triangle, and rectangle represent the CG, CHG and CHH contexts. The filled red, green and blue cycles represent methylated and the unfilled cycle, triangle, and rectangle represent unmethylated cytosine sites

We selected the AGO1d (TraesCS7A02G557400)- *and* H2A3*-*encoding genes (TraesCS1B02G048900) for expression and methylation level validation. Four sites were chosen for validation of the methylation level in BS366 under cold and control conditions using bisulfite sequencing. The expression and methylation levels correlated well with those in the sequencing data. The expression of the *AGO1d* gene was higher under cold conditions (Fig. 11a), but four CG sites in the gene were found to be hypermethylated in BS366 under control conditions in the *AGO1d* gene (Fig. 11b and c). For the H2A3-encoding gene, the expression level was higher in BS366 under cold conditions, but the methylation level was higher in the CG, CHG, and CHH contexts (Fig. 12a, b). As shown in Fig. 12c, four CG sites were hypermethylated in the control condition compared with the cold condition. Other validated DMSs are shown in Additional file 1: Figs. S7, S8, and S9, all of which correlated well with the MethylRAD sequencing data.

## Discussion

Known as the prerequisite component for the heterosis breeding system, the male sterile line directly determined the hybrid seed purity and yield. Therefore, a deep understanding of wheat sterility and the mechanisms and gene networks that lead to male sterility is needed. In this study, a TGMS line (BS366) and normal inbred lines (J411) were used to explore the underlying male sterile mechanisms. BS366 were sterile under cold condition. They had smaller anther and sterile pollens with no starch accumulation. Histological observation revealed the unusual formation of dyads and tetrads during meiosis and of vacuolated stage pollen. All of these factors led to male sterility of BS366 pollen under cold conditions. Transcriptome and reduced methylome sequencing were carried out for BS366 and J411 under cold and control conditions. All the DEGs and DMSs were identified, and pathways involved in cell division and carbohydrate and lipid metabolism were found to be correlated with BS366 male sterility. We also found that carbohydrate transport, oxylipin biosynthetic process, positive regulation of transcription, and lipid transport were differentially methylated in BS366 under cold and control conditions.

### Cell division was impaired in BS366 at anaphase in meiosis I and II

During cytokinesis in plants, the parent cell divides into two daughter cells via physical insertion of a membranous cell plate, which involves de novo construction of a cell wall [21, 39, 40]. The phragmoplast, known as a plant-specific cytoskeletal configuration, is involved in cell wall assembly at late anaphase [41]. Phragmoplasts in plants are composed of microtubules (MTs), microfilaments, motor proteins, and several regulators [41–45]. MTs in phragmoplasts are organized as two opposite sets, which overlap at the equator [46, 47]. Golgi-derived vesicles are responsible for the transportation and fusion of cell wall-containing materials to this site to form the cell plate [22]. Finally, the cell plate assembles toward the cell edges in an actin-dependent process [48, 49].

Histological observation indicated disrupted dynamic organization of phragmoplast microtubules and deposition of the cell plate, causing defective cytokinesis during meiosis I [50]. The BS366- and BS366-specific DEGs were enriched in biological processes, including microtubule-based processes, cytokinesis by cell plate formation, cytoskeleton-dependent cytokinesis, and microtubule-based movement. As indicated above, phragmoplast-initiated cell plate formation was impaired in this study (Figs. 2 and 3). The significant enrichment of those processes corresponded well to the histological observations. We further inspected the DEGs involved in those processes and found that the genes included tubulin-related proteins, kinesin-related protein-encoding genes, MT-associated protein 65 (MAP65)-related protein-encoding genes, and MCM-related genes (Additional file 2: Table S6). MTs are assembled by heterodimers of alpha- and beta-tubulin GTPases in a head-to-tail manner, which serve as tracks for transport and frameworks for spindle assembly and phragmoplast formation [51, 52]. Studies involved in genetic analysis have shown that β-tubulin is essential for MT organization in *A. thaliana* [53, 54]. We also found that seven α-tubulin- and eight β-tubulin-encoding genes were differentially expressed between BS366_col and BS366_con, all of which were expressed at higher levels in BS366_col than in BS366_con (Additional file 2: Table S6). In this study, the abnormal expression pattern of these tubulin genes indicated that the structure and trafficking in MTs might be impaired by low temperature in BS366 spikes.

During cytokinesis, cell plate biogenesis is accompanied by vesicle fusion at its margins and the dynamic turnover of microtubules [55]. This rapid turnover in plant cells must be initiated by MT-associated proteins (MAPs) [41]. In plant cytokinesis, MAP65 contributes to the stabilization of antiparallel microtubule overlaps in the phragmoplast [56, 57]. MAP65–3 plays a critical role in organizing the mitotic microtubule array during both early and late mitosis in all plant organs [58]. In this study, we screened all the orthologues of the nine MAP65 family genes in *Arabidopsis thaliana* [59] and identified 30 orthologous genes encoding MAP65-related proteins. Among those genes, two (TraesCS7A02G223100, TraesCS7B02G190000) encoding orthologs of MAP65–1, one (TraesCS7D02G224800) encoding an ortholog of MAP65–2 and one (TraesCS3A02G264600) encoding an ortholog of MAP65–3 in *Arabidopsis* were differentially expressed between BS366_col and BS366_con. All four genes were expressed at higher levels under cold than under control conditions (Additional file 2: Table S7). Kinesins were the largest cytoskeletal protein family in flowering plants. Cytoskeleton-based motors use the energy of release from ATP hydrolysis to move along MT tracks [60]. In *Arabidopsis thaliana*, Kinesin-12A and Kinesin-12B collaboratively play a critical role in the organization of phragmoplast microtubules. In the double mutant, the first postmeiotic cytokinesis was abolished without the formation of a cell plate [61]. *OsKCH2* encodes a plant-specific kinesin-14 with an N-terminal actin-binding domain and a central motor domain. It specifically decorates preprophase band microtubules in vivo and transports actin filaments along microtubules in vitro [62]. In this study, 12 genes encoding kinesin-related proteins were found to be differentially expressed in BS366 cells between cold and control conditions. Most of these genes were induced in BS366_col (Additional file 2: Table S7). We may conclude that lower temperature changed the expression of genes involved in cell plate formation, which finally resulted in male sterility in BS366 spikes.

### Carbohydrate and lipid metabolism pathways were altered in BS366 under cold conditions

One reason for male sterility in BS366 was that the abnormal dyads and tetrads cannot successfully enter the development of microspores because they were unable to generate uninucleate pollen grains; the other reason was the abnormality in microspore development. As shown in Figs. 2 and 3, the pollen grains in sterile BS366 shrank at the vacuolated stage. No starch accumulated in the sterile compared with the fertile pollen.

Carbohydrates are known to play important roles in anther development by serving as nutrients and signals. Anthers cannot synthesize photosynthetic assimilates themselves [63]. Pollen develops inside the anther by immersion in locular fluid, which provides lipids and sugars generated from the degradation of the tapetum [64]. At the late gametogenesis stage, the pollen matured with a sign of starch accumulation, which functions as energy for seed germination [65]. In this study, genes involved in carbohydrate metabolism and glycan biosynthesis and metabolism were exclusively differentially expressed between BS366_col and BS366_con. Genes encoding proteins involved in carbohydrate metabolism and transport, such as ADP-glucose pyrophosphorylase large subunit, beta-galactosidase 5, callose synthase 10, callose synthase 3, putative cellulose synthase A catalytic subunit 1 (UDP-forming), alpha-1,4-glucan-protein synthase (UDP-forming), sucrose: fructan 6-fructosyltransferase, sucrose-phosphate synthase 9 putative xyloglucan endotransglucosylase/hydrolase protein 23, sugar transport protein 14, UDP-glycosyltransferase 85A2, and xylan arabinosyl transferase, were differentially expressed in BS366 between cold and control conditions (Additional file 2: Table S8).

Plant anthers are multilayered, multifunctional tissue. The tapetum provides sporopollenin and pollen coat constituents generated by lipid metabolism, which is essential for exine formation [17]. Sporopollenin precursors, cutin, and wax are synthesized in the tapetum and translocated into the locule by ABCG transporters or lipid transport proteins to facilitate anther cuticle and pollen exine development [66]. In rice and *Arabidopsis*, genes involved in the translocation of lipids have been identified. It has been reported that ATP-binding cassette transport protein (ABCG15) and nonspecific lipid transfer proteins (nsLTPs) function in sporopollenin precursor transportation [67, 68]. ABCG26 is involved in transporting components of sporopollenin and spermidines into the anther locule prior to tapetum degradation [69]. Another analysis in *Arabidopsis* has shown that III-LTPs are involved in allocating and incorporating lipidic compounds into the pollen wall [70]. More recently, a wheat gene termed *TaMs1*, encoding a glycosylphophatidylinositol (GPI) LTP, was demonstrated to be required for wheat male fertility [11, 71]. It has been reported that male fertility in *Arabidopsis* is also influenced by jasmonates, fatty acid-derived products catalyzed by 13-lipoxygenases (13-LOXs). Lipoxygenase 2 (LOX2) is dispensable for fertility. The double mutant *lox3lox4* is male sterile and shows indehiscent anthers and sterile pollen grains [72]. In the present study, two LOX2, one LOX3, and putative lipoyltransferase-like protein-encoding genes were identified among the BS366-specific DEGs (Additional file 2: Table S8).

The BS366-specific DEGs were assigned to metabolic pathways, such as lipid metabolism. As indicated by the DNA methylation analysis, genes involved in lipid metabolism, including the oxylipin biosynthetic process and lipid transport, were differentially methylated. The KEGG analysis also revealed significantly represented pathways, such as the phospholipase D signaling pathway, fatty acid biosynthesis, and fatty acid degradation (Fig. 9). Several genes involved in the transport of lipids or their derivatives were identified, including five ABC transporters, two nonspecific lipid transfer proteins (nsLTPs), and one gene homolog to *ABCG26* in *Arabidopsis*. Taken together, results indicated that lipid metabolism and transport might be impaired by low temperature, which caused male sterility in BS366 spikes (Additional file 2: Table S8).

### DNA methylation may be involved in male sterility in BS366

The development of reproductive organs directly determines crop yield. Thus, understanding the manner of reproductive organs in response to temperature changes is of great importance. The male reproductive organs were more vulnerable to temperature damage than the female reproductive and other organs, especially during the flowering stage and the young microspore stage [63, 73]. It has been reported that epigenetic regulation is involved in transcriptional regulation in response to abiotic stress, such as temperature challenges [74]. The DNA methylation level in the normal cotton line was induced by high temperature (HT) but repressed in the HT-sensitive cotton line [30]. Hypermethylation has been observed in the PTGMS line PA64S at a temperature higher than 23.5 °C under long-day conditions [37]. In this study, the expression profile showed that processes related to DNA methylation were significantly enriched. Several *DRM1-* or *DRM1*-related genes were exclusively differentially expressed between cold and control conditions in BS366 and assigned to the turquoise module (Supplementary Fig. S3). These *DRM1* genes were all expressed at higher levels in BS366_col than in fertile BS366. Thus, we could conclude that the temperature-induced expression of DNA methylation genes might be involved in male sterility in BS366 spikes. In the Methyl-RAD sequencing, differentially methylated sites were located in genes assigned to the phospholipase D signaling pathway, fatty acid biosynthesis, and fatty acid degradation (Fig. 9b). In the GO analysis of DMSs specific for BS366, biological processes including carbohydrate transport, oxylipin biosynthetic process, and lipid transport were also significantly enriched (Supplementary Table S6). Pollen wall development after the release of microspores from tetrads has been reported to require the involvement of fatty acid and lipid metabolism pathways. Taken together, these results suggest that DNA methylation may be involved in male sterility in BS366 spikes under cold conditions through induced expression of DNA methyltransferase and suppression of fatty acid and lipid metabolism pathways.

## Conclusions

Sterile BS366 has smaller anther and sterile pollens with no starch accumulation. Histological observation revealed that the formation of dyads and tetrads during meiosis and vacuolated stage pollen were abnormal. Compared with fertile BS366, genes involved in biological processes, including meiotic sister chromatid segregation and cohesion, mRNA cleavage involved in gene silencing, and microtubule-based movemen were down-regulated, while genes involved in DNA methylation and histone modification were up-regulated in the sterile BS366. DNA methylation sequencing revealed that the methylation level involved in carbohydrate transport, fatty acid metabolism, and lipid transport was altered between sterile and fertile BS366 spikes. These results provide insights into the DNA methylation involved in temperature-sensitive genic male sterility in BS366 spikes.

## Methods

### Plant materials

The wheat temperature-sensitive genic male sterile (TGMS) line BS366 and the normal inbred line Jing411 (J411), both maintained at Beijing Engineering Research Center for Hybrid Wheat, were used in this study. One hundred fifty seeds for BS366 or J411 were planted in 15 plastic pots evenly in early October. The plastic pots were embedded in the ground in early October 2016 at the experimental farm and moved into the greenhouse after natural vernalization. The natural vernalization needs 40 days with an average temperature below 4 °C. At least five individuals were kept for each pot. Before the five-leaf stage, ten pots of BS366 or J411 plants of uniform growth were selected and then randomly assigned to the cold and control temperature groups. The selected plants were grown in phytotrons (Koito, Tokyo, Japan) at 20 °C with a 12-h photoperiod to control the temperature for the entire reproductive period. Cold temperature treatment was implemented with a temperature of 10 °C and a 12-h photoperiod for 10 days and then transferred to the control temperature environment for the entire reproductive period.

### Phenotypic analysis of BS366

Photographs of the flower tissue for BS366 under cold and control environments were obtained using ZEISS SteREO Discovery, V20. To evaluate pollen viability, cold and control anthers were separately crushed, stained with 1% iodine-potassium iodide (I_2_-KI) solution and photographed under an Olympus BX-53 microscope (Tokyo, Japan). For microspore and anther phenotype observation, anthers and spikelets from the corresponding developmental stages of BS366, from meiosis to the mature pollen stage under both control and cold conditions, were collected and fixed in FAA solution (formaldehyde: glacial acetic acid: 50% ethanol =5: 5: 9). The anthers were separated from the young spikes, mashed with tweezers to release the pollen, and dyed with improved carbol fuchsin solution. Photographs of microspores and pollen were obtained using an Olympus BX-53 microscope (Tokyo, Japan). For the anther phenotype analysis, the anthers were fixed in FAA solution, removed from the FAA fixative, dehydrated in an ethanol series, and then embedded in paraffin. Tissue sections were cut transversely from the wax-embedded anthers and stained using safranin O-fast green. The anther morphology was analyzed with a scanning electron microscope (HITACHI SU8100).

### Sample preparation, RNA isolation and real-time qRT-PCR

Among the 25 plants assigned to cold or control conditions, we chose 15 plants of uniform growth for sampling. Three main spikes of BS366 or J411 in the control or cold conditions from the meiosis to the vacuolated stage were pooled together with two replicates. All samples were immediately frozen in liquid nitrogen and stored at − 80 °C for RNA extraction. Total RNA from spikes of both lines under cold and control conditions was extracted using TRIzol Reagent (Invitrogen Corp., Carlsbad, CA). The concentration and quality of total RNA were determined with a Nanodrop spectrophotometer and 1% agarose gel electrophoresis and subjected to transcriptome sequencing. For real-time qRT-PCR, cDNA was synthesized using the PrimeScript™ RT reagent Kit with gDNA Eraser (Takara). Differentially expressed genes were validated with a CFX96 Touch™ Real-Time PCR Detection System (Bio-Rad Laboratories, Hercules, CA, USA) using SYBR Green II (Takara). The expression levels of genes in samples were normalized using the endogenous wheat 18S gene with primer sequences 5′-TGCTGGAATCGGAATAGTTGAG-3′ and 5′-ACTACGCAGGCTCATCAAACAG-3′. The relative expression levels were calculated using the 2^−ΔΔCt^ method. Primer sequences were designed using Primer3 input version 4.0.0 (http://primer3.ut.ee/) and are listed in Additional file 2: Table S9.

### Transcriptome sequencing and data analysis

All samples were sequenced using the Illumina HiSeq 2500 platform. Raw reads were filtered to remove low-quality reads containing more than 30% bases with a Q-value < 20. After trimming low-quality bases (Q-value < 20) from the 5′ and 3′ ends of the remaining reads, the resulting high-quality clean reads in each sample were mapped to the wheat reference genome (IWGSC RefSeq v1.0) using HISAT (v2.0.6) [75]. Only reads that were uniquely mapped to the reference genome were kept for further analysis. Fragments per kilobase of exon model per million mapped reads (FPKM) was used to estimate transcript expression levels. Differentially expressed genes (DEGs) in each comparison were identified by DEseq2 using a threshold *P* value < 0.05 and a fold change ≥2 [76]. The identified DEGs were subjected to Gene Ontology (GO) [77] and Kyoto Encyclopedia of Genes and Genomes (KEGG) analyses [78] as previously described.

To analyze the influence of the power value on the scale independence and mean connectivity, we used the function softConnectivity from the package of Weighted gene coexpression network analysis (WGCNA), with the “randomly selected genes” parameter set at 5000, other parameters set as default, and the power parameter precalculated by the pickSoftThreshold function in WGCNA. This function provides the appropriate soft-thresholding power for network construction by calculating the scale-free topology fit index for several powers. We next summarized the expression values using the function collapseRows implemented in the R package WGCNA. Cluster analysis was subsequently performed by flashClust [79].

### DNA sample isolation, MethylRAD library preparation and sequencing

The wheat spikes of BS366 and J411 of control and cold conditions were sampled using the sampling method used for the transcriptome sequencing. Genomic DNA was extracted from spike tissues using the cetyltrimethyl ammonium bromide method. The MethylRAD library was prepared by digesting genomic DNA using FspEI (New England Biolabs, Ipswich, MA, USA) at 37 °C for 45 min. The digested products were verified on a 1% agarose gel. Then, the digested DNA was ligated to adaptor A and adaptor B using T4 DNA ligase (New England Biolabs, Ipswich, MA, USA). The ligation products were amplified using Phusion high-fidelity DNA polymerase (New England Biolabs, Ipswich, MA, USA). PCR was conducted using a MyCycler thermal cycler (Bio-Rad) with 16 cycles of 98 °C for 5 s, 60 °C for 20 s, 72 °C for 10 s, and a final extension of 5 min at 72 °C. The target band (approx. 100 bp) was excised from an 8% polyacrylamide gel and diffused from the gel in nuclease-free water for 30 min at 37 °C. DNA was PCR amplified as described above using 4–6 cycles. After purifying the PCR products using a MinElute PCR Purification Kit (Qiagen), the barcodes were introduced by means of PCR using Phusion high-fidelity DNA polymerase (2 U/μl) (New England Biolabs, Ipswich, MA, USA). The PCR products were purified using a MinElute PCR Purification Kit (Qiagen) and subjected to paired-end sequencing (100–150 bp) on an Illumina HiSeq X Ten platform.

### DNA methylation data analysis

Raw reads were first trimmed to remove adaptor sequences. Reads containing ambiguous base calls (N) or an excessive number of low-quality bases (more than five bases with a quality less than 10) were removed. The high-quality reads were used for subsequent analysis. The Methyl-RAD sequencing tags for each sample were mapped to the reference genome (IWGSC RefSeq v1.0) using Bowtie2 software (version 2.3.4.1) [80]. The number of reads was normalized as RPM (reads per million) to quantify the methylation level of all the methylated sites. Differentially methylated sites (DMSs) were identified using DEseq with a threshold fold change greater than 2 and a *P* value less than 0.05 [76]. Genes containing differentially methylated loci were subjected to Gene Ontology (GO) and Kyoto Encyclopedia of Genes and Genomes (KEGG) analysis [77, 78].

### Pyrosequencing methylation analysis

We selected the candidate genes that resulted from combining the transcriptome and MethylRAD sequencing data. The sequence of the differentially methylated region was subjected to pyrosequencing methylation sequencing. Primers for the target site were designed using PyroMark Assay Design 2.0 (Qiagen). All the primer sequences are listed in Additional file 2: Table S9. Genomic DNA was first modified with sodium bisulfite to convert the unmethylated Cs into Ts with the EpiTect Bisulfite kit (Qiagen, Germany) following the manufacturer’s instructions. The modified DNA was purified using MinElute DNA spin columns (Qiagen, Germany). For each PCR, 1.0 μl of bisulfite-treated DNA was used in a 50-μl reaction system. These PCR products were gel-purified using a gel extraction kit (Omega, USA), cloned into the pMD18-T vector (Takara, Dalian, China) and sequenced. At least ten clones were sequenced for each sample.

## Supplementary Information

**Additional file 1.**

**Additional file 2.**

## Data Availability

Transcriptome and methylation sequence data for all samples can be found in the National Genomics Data Center (https://bigd.big.ac.cn/) under accession number CRA003366.
