# Supplementary material for: Comparative transcriptome and DNA methylation analysis in temperature-sensitive genic male sterile wheat BS366
Source: BMC Genomics. 2021 Dec 20;22:911. doi: 10.1186/s12864-021-08163-3 (PMC8686610; doi:10.1186/s12864-021-08163-3)
Supplement: Supplementary file 1 — Additional file 1. [file 12864_2021_8163_MOESM1_ESM.docx]

**Supplementary Figures to**

**Comparative transcriptome and DNA methylation analysis in temperature-sensitive genic male sterile wheat BS366**

Yong-jie Liu^1,2*^, Dan Li^1,2*^, Jie Gong^1,2^, Yong-bo Wang^1^, Zhao-bo Chen^1^, Bin-shuang Pang^1,2^, Xian-chao Chen^1^, Jian-gang Gao^1^, Wei-bing Yang^1^, Feng-ting Zhang^1＆^, Yi-miao Tang^1,2＆^, Chang-ping Zhao^1,2＆^, Shi-qing Gao^1,2＆^

^1^Beijing Engineering Research Center for Hybrid Wheat, Beijing Academy of Agriculture and Forestry Sciences, Beijing, 100097, China

^2^The Municipal Key Laboratory of the Molecular Genetics of Hybrid Wheat, 100097, China

^＆^Corresponding authors: gshiq@126.com; [cp_zhao@vip.sohu.com](mailto:cp_zhao@vip.sohu.com), tangyimiao@126.com; lyezh@163.com.

Email:

Shi-qing Gao: gaoshiqing@baafs.net.cn; gshiq@126.com

Chang-ping Zhao: zhaochangping@baafs.net.cn; cp_zhao@vip.sohu.com

^*^Coauthors: Yong-jie Liu and Dan Li contributed equally to this work.

^＆^Corresponding authors: gaoshiqing@baafs.net.cn; zhaochangping@baafs.net.cn; tangyimiao@126.com; lyezh@163.com.

*
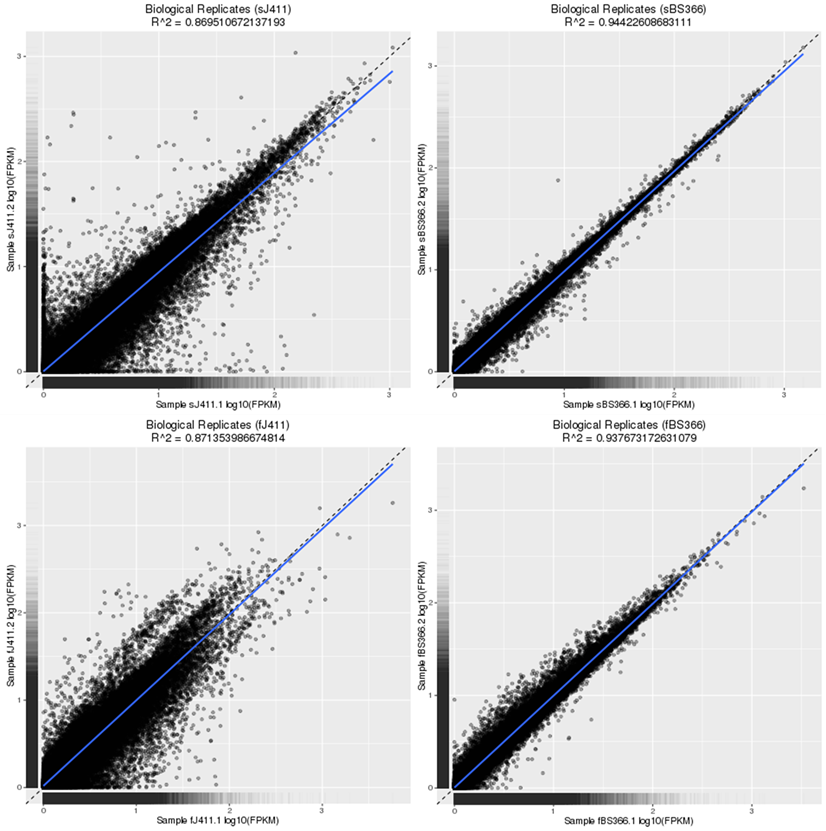
*

**Fig S1** The correlation between biological replicates for BS366 and J411 under cold and control conditions.


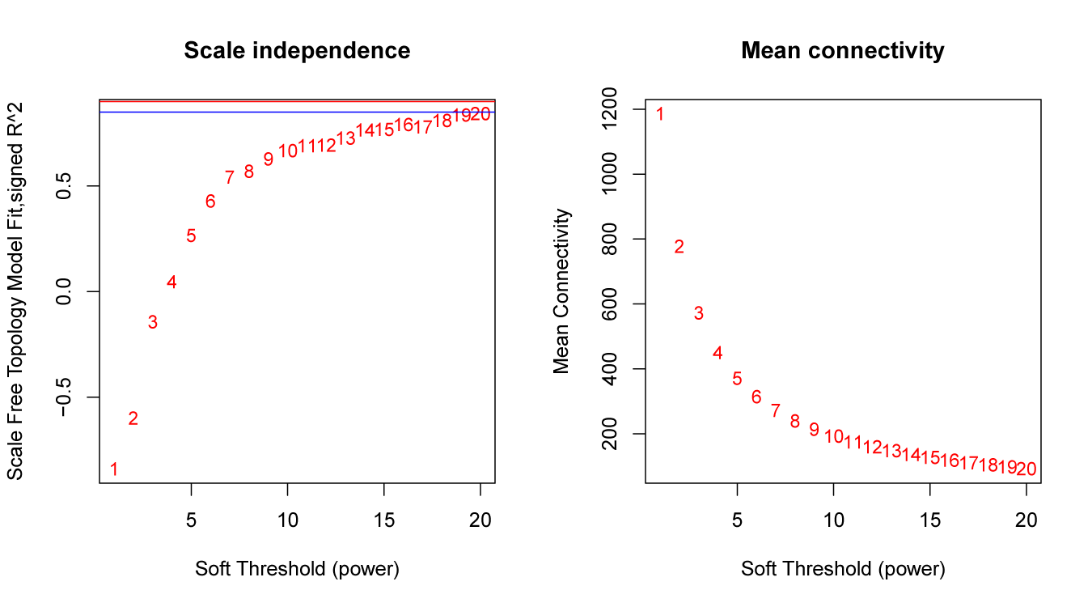


**Fig S2** Analysis of network topology for various soft-thresholding powers. The left panel shows the scale-free fit index(*y*-axis) as a function of the soft-thresholding power (*x*-axis). The right panel displays the mean connectivity (degree, *y*-axis) as a function of the soft-thresholding power (*x*-axis)

**
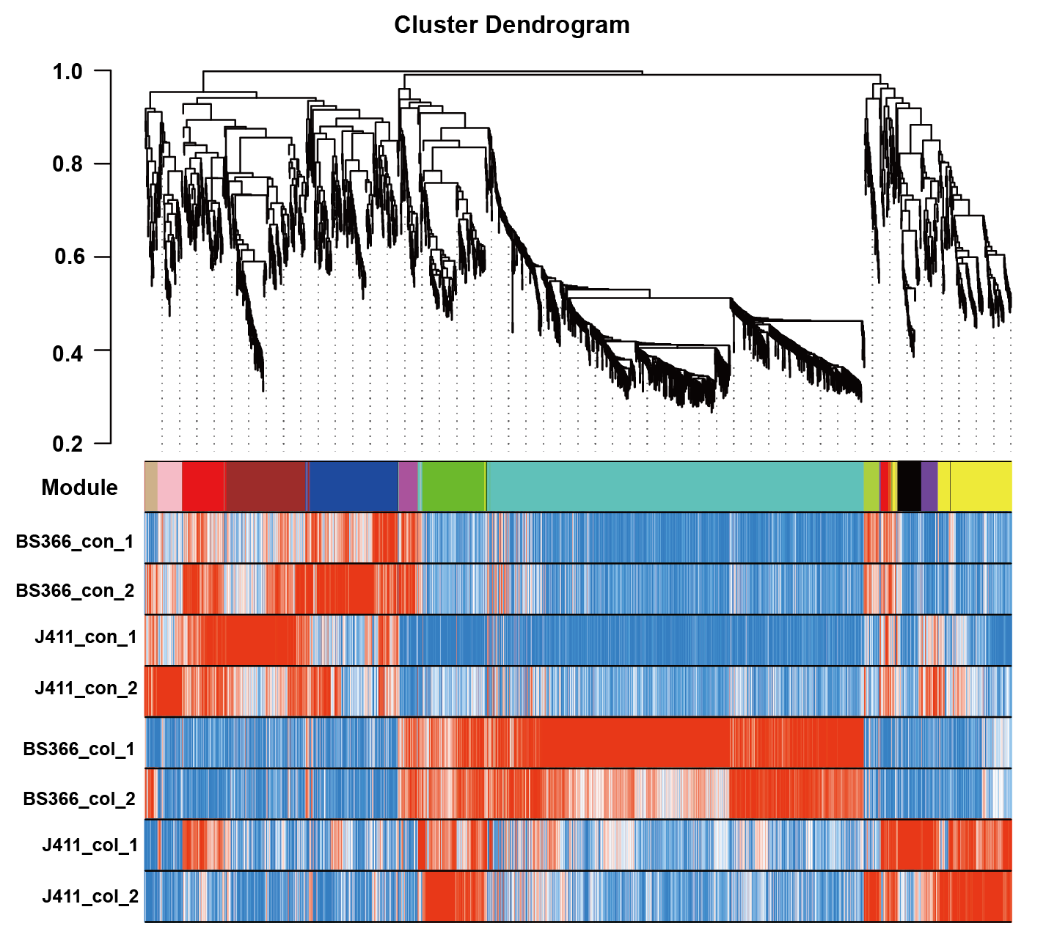
**

**Fig S3** Network analysis dendrogram showing modules identified by weighted gene co-expression network analysis (WGCNA). Clustering dendrogram of genes, with dissimilarity based on the topological overlap, together with assigned module colors. Blue indicates lower expression and red indicate higher expression level. As a result, 12 co-expression modules were constructed and was shown in a different color. These modules were ranged from large to small by the number of genes they included


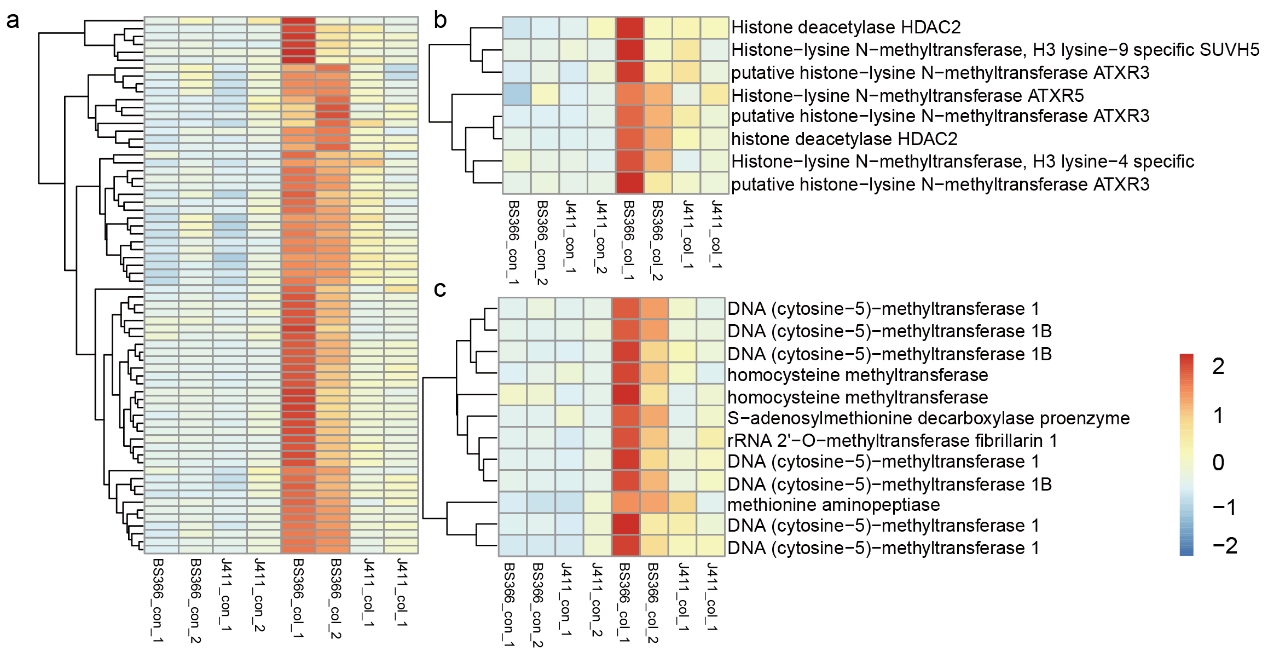


**Fig S4** Expression patterns of genes involved in histone constitution, DNA methylation, and histone modification. **a** expression pattern of histone constitution genes, including Histone H1, Histone H2A, Histone H2B, Histone H3. **b** Expression pattern of Histone modification-related genes. **c** Expression pattern of DNA methyltransferase genes. Red color indicates a high expression level and blue indicate a low expression level


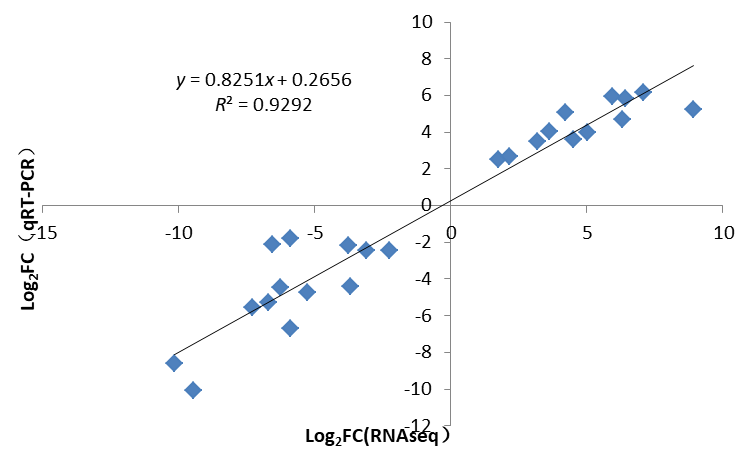


**Fig S5** The real-time qRT-PCR validation of the differentially expressed genes.


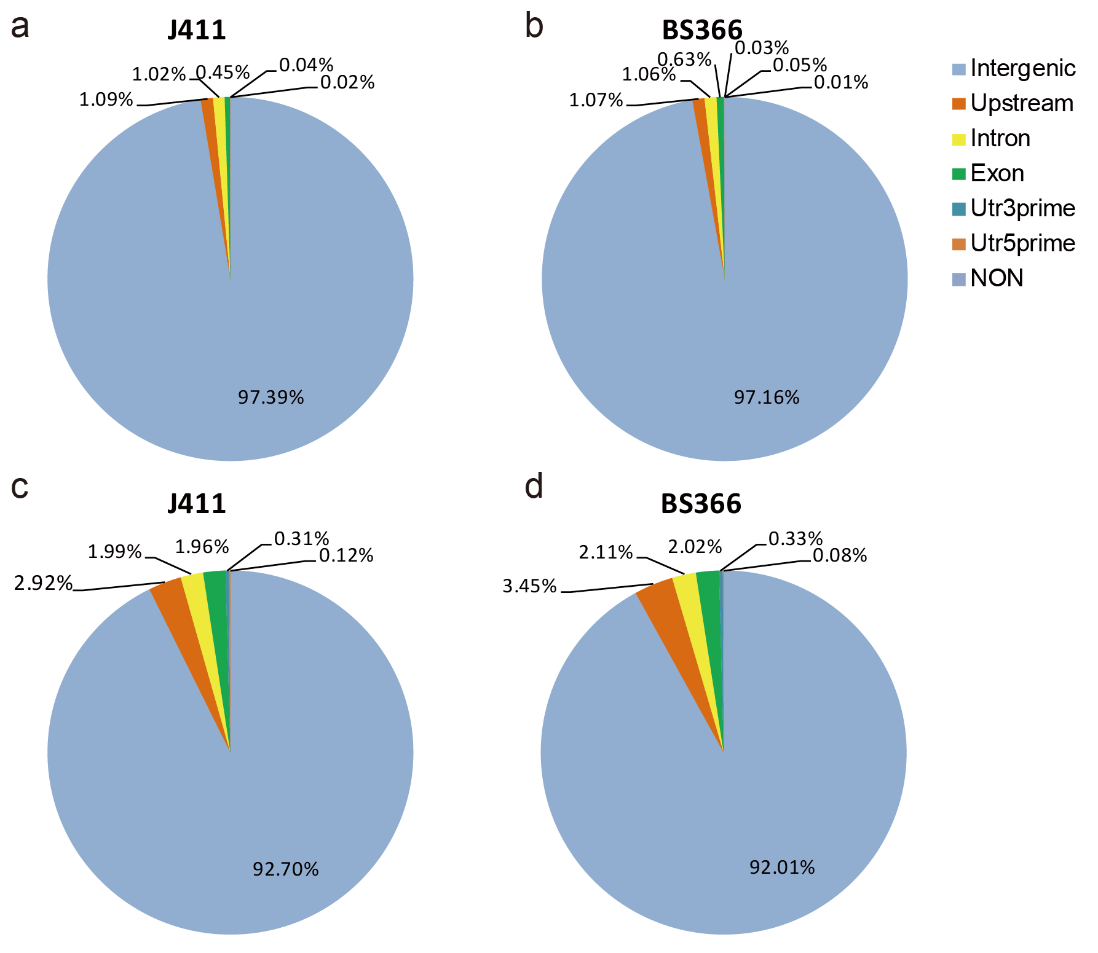


**Fig S6** Distribution of DMSs in the genome elements. The distribution of DMSs in J411(**a**) and BS366 (**b**) under control and J411(**c**) and BS366 (**d**) under cold conditions


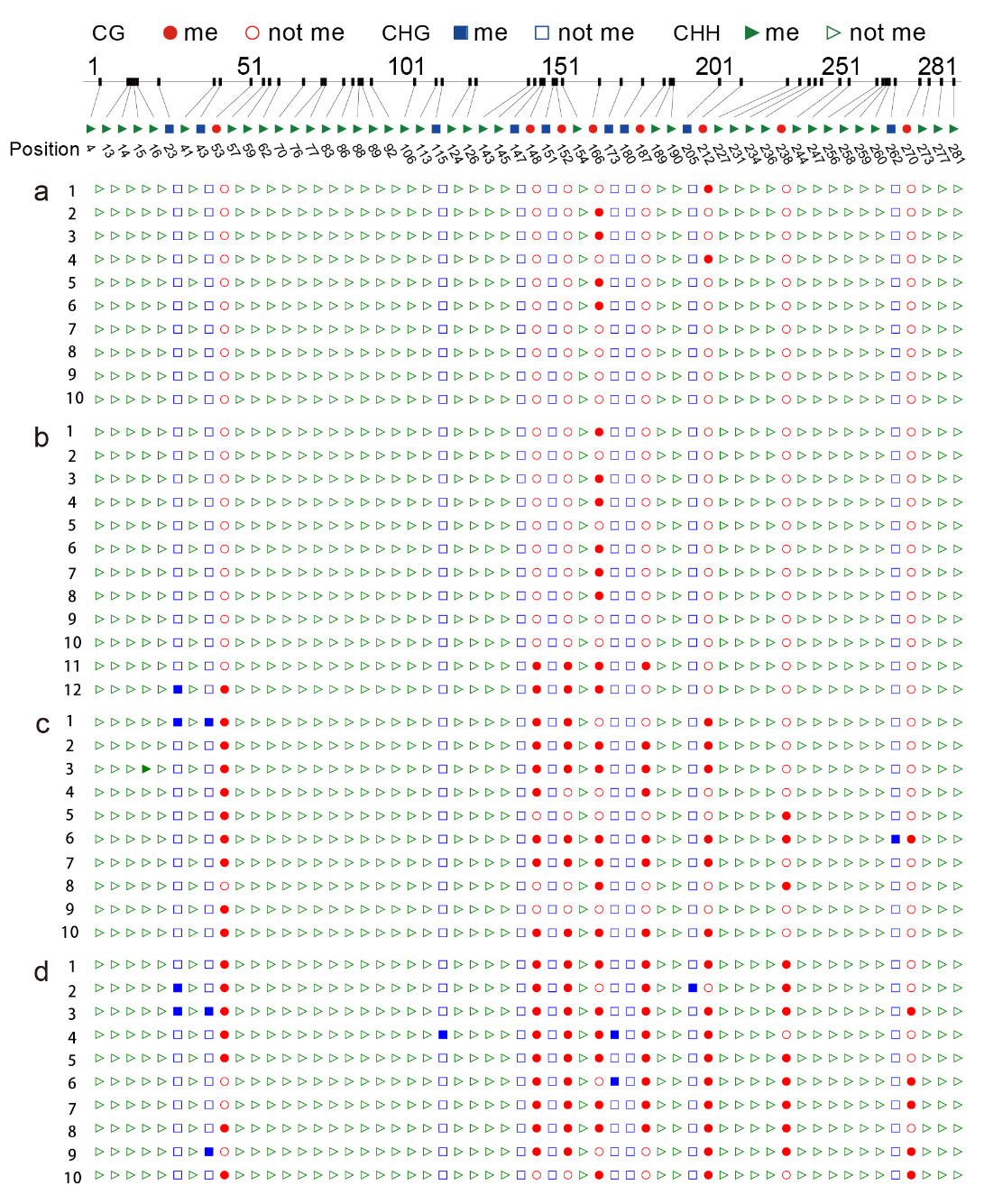


**Fig S7** Pyrosequencing methylation validation of TraesCSU02G000100 in BS366 between the cold and control conditions. The distribution of CG, CHG, and CHH for two replicates of BS366 under cold (**a** and **b**) and control (**c** and **d**) conditions. The cycle, triangle, and rectangle represent CG, CHG and CHH context. The red, green, and blue filled represent methylated cytosine, and the unfilled dot, triangle and rectangle represents the unmethylated cytosine sites

**
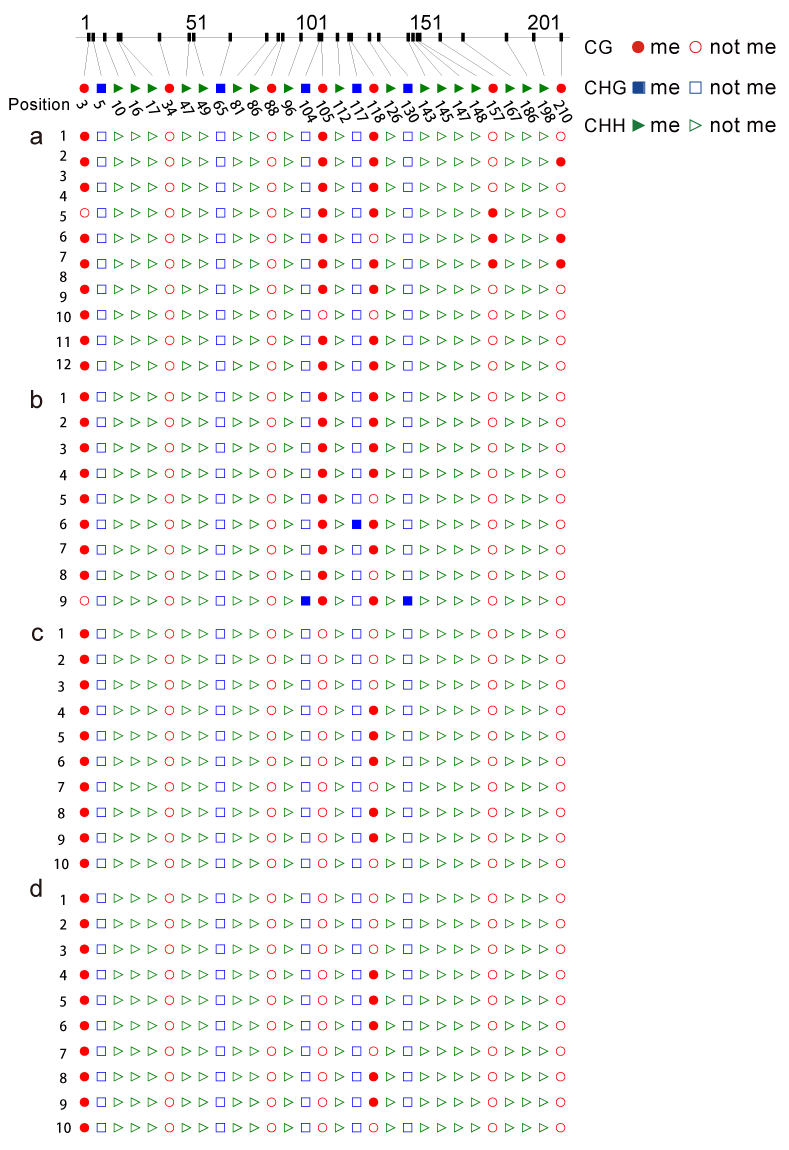
**

**Fig S8** Pyrosequencing methylation validation of TraesCS5A02G086600 in BS366 between the cold and control conditions. The distribution of CG, CHG, and CHH for two replicates of BS366 under cold (**a** and **b**) and control (**c** and **d**) conditions. The cycle, triangle, and rectangle represent CG, CHG and CHH context. The red, green, and blue filled represent methylated cytosine, and the unfilled dot, triangle and rectangle represents the unmethylated cytosine sites


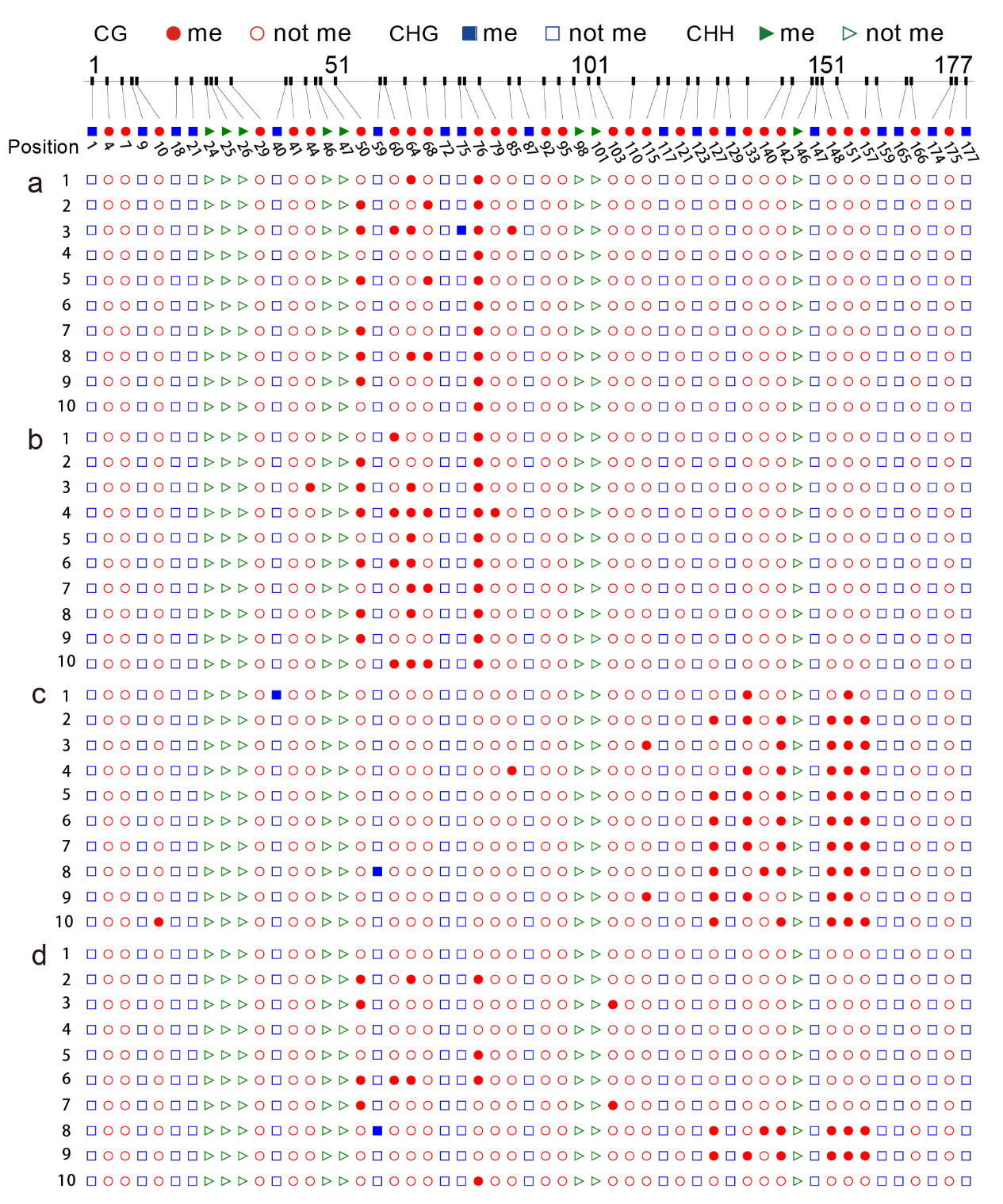


**Fig S9** Pyrosequencing methylation validation of TraesCS7D02G161000 in BS366 between the cold and control conditions. The distribution of CG, CHG, and CHH for two replicates of BS366 under cold (**a** and **b**) and control (**c** and **d**) conditions. The cycle, triangle, and rectangle represent CG, CHG and CHH context. The red, green, and blue filled represent methylated cytosine, and the unfilled dot, triangle and rectangle represents the unmethylated cytosine sites
